# Discovery of novel geranylgeranyl reductases and characterization of their substrate promiscuity

**DOI:** 10.1186/s13068-018-1342-2

**Published:** 2018-12-28

**Authors:** Corey W. Meadows, Florence Mingardon, Brett M. Garabedian, Edward E. K. Baidoo, Veronica T. Benites, Andria V. Rodrigues, Raya Abourjeily, Angelique Chanal, Taek Soon Lee

**Affiliations:** 1 Joint BioEnergy Institute, 5885 Hollis Street, 4th floor, Emeryville, CA 94608 USA; 2 Biological Systems & Engineering Division, Lawrence Berkeley National Laboratory, Berkeley, CA 94720 USA; 3 Total Raffinage Chimie, 2 Pl. Jean Millier, 92400 Courbevoie, France

**Keywords:** Geranylgeranyl reductase, Redox enzymes, Isoprenoids, Hydrogenation, Terpene biosynthesis

## Abstract

**Background:**

Geranylgeranyl reductase (GGR) is a flavin-containing redox enzyme that hydrogenates a variety of unactivated polyprenyl substrates, which are further processed mostly for lipid biosynthesis in archaea or chlorophyll biosynthesis in plants. To date, only a few GGR genes have been confirmed to reduce polyprenyl substrates in vitro or in vivo.

**Results:**

In this work, we aimed to expand the confirmed GGR activity space by searching for novel genes that function under amenable conditions for microbial mesophilic growth in conventional hosts such as *Escherichia coli* or *Saccharomyces cerevisiae.* 31 putative GGRs were selected to test for potential reductase activity in vitro on farnesyl pyrophosphate, geranylgeranyl pyrophosphate, farnesol (FOH), and geranylgeraniol (GGOH). We report the discovery of several novel GGRs exhibiting significant activity toward various polyprenyl substrates under mild conditions (i.e., pH 7.4, *T* = 37 °C), including the discovery of a novel bacterial GGR isolated from *Streptomyces coelicolor*. In addition, we uncover new mechanistic insights within several GGR variants, including GGR-mediated phosphatase activity toward polyprenyl pyrophosphates and the first demonstration of completely hydrogenated GGOH and FOH substrates.

**Conclusion:**

These collective results enhance the potential for metabolic engineers to manufacture a variety of isoprenoid-based biofuels, polymers, and chemical feedstocks in common microbial hosts such as *E. coli* or *S. cerevisiae.*

**Electronic supplementary material:**

The online version of this article (10.1186/s13068-018-1342-2) contains supplementary material, which is available to authorized users.

## Background

Manufacturing of terpenoid-based compounds has been studied extensively in synthetic biology. The two biosynthetic pathways for terpene monomer biosynthesis are the mevalonate and 1-deoxy-d-xylulose 5-phosphate pathways, where pyruvate is ultimately converted into either of the C_5_ terpene building blocks, isopentenyl pyrophosphate or dimethylallyl pyrophosphate [1, 2]. These monomer units are subsequently fused by various prenyl transferases to make geranyl pyrophosphate (GPP, C_10_), farnesyl pyrophosphate (FPP, C_15_), and geranylgeranyl pyrophosphate (GGPP, C_20_) [3]. The structural diversity of terpenes allows for a broad range of uses in areas including dietary supplements, polymer feedstocks, pharmaceuticals and cosmetics, household cleaners, and fuels [4–8]. Much of this structural diversity is achieved via downstream cyclization and redox steps on GPP, FPP, and GGPP using a plethora of terpene synthases [9–11]. Combinations of these core isoprenoid pyrophosphate intermediates serve as starting points for cholesterol biosynthesis, antibiotic biosynthesis, cofactor biosynthesis, and protein prenylation [12–16].

While microbes including *E. coli* and *S. cerevisiae* have emerged as robust hosts in the production of terpenoids, producing specially tailored natural products will require the use of novel chemistries and biosynthetic pathways. For example, isoprenoids have been considered as a promising precursor of alternative fuels, but reduction of isoprenoid double bonds is required to decrease the reactivity and sensitivity to oxidation and make them better fuels. Enzymatic alkene hydrogenation, however, is typically assisted by adjacent electron-withdrawing groups as observed in examples including old yellow enzyme, fatty acid enoyl reductases, and enone reductases [17–20].

Reduction of unactivated substrates like prenyl pyrophosphates typically involves oxidoreductases from the geranylgeranyl reductase (GGR) family. GGR generates fully saturated isoprenoid intermediates in archaeal membrane biosynthesis [21, 22]. In archaea, GGR’s native activity is believed to fully reduce all prenyl groups within the C_20_ isoprenoid chain of 2,3-di-*O*-geranylgeranylglyceryl phosphate (DGGGP) before carbon–carbon bond formation of reduced C_20_ isoprenoid chains form fully reduced C_40_ precursors needed for membrane synthesis [23, 24]. Moreover, in various organisms such as eukaryotes, bacteria, and archaea, GGRs also have been demonstrated to reduce a variety of prenylated substrates, including chlorophyll, tocopherol, dolichol, and menaquinone [25–28]. However, very few GGRs have been confirmed as oxidoreductases, and most enzymes having prenyl reductase activity were derived from species that thrive under extremophilic conditions or utilize photosynthesis for energy transduction [25–32]. To date, only two crystal structures have been solved for GGRs from archaeal organisms. Reducing equivalents are thought to be derived from a NAD(P)H/ferredoxin reductase, in which electron transfer is conducted throughout the protein and modulated by a conserved active site cysteine within the cofactor binding domain, located directly behind the FAD isoalloxazine ring [31].

**Scheme 1:**
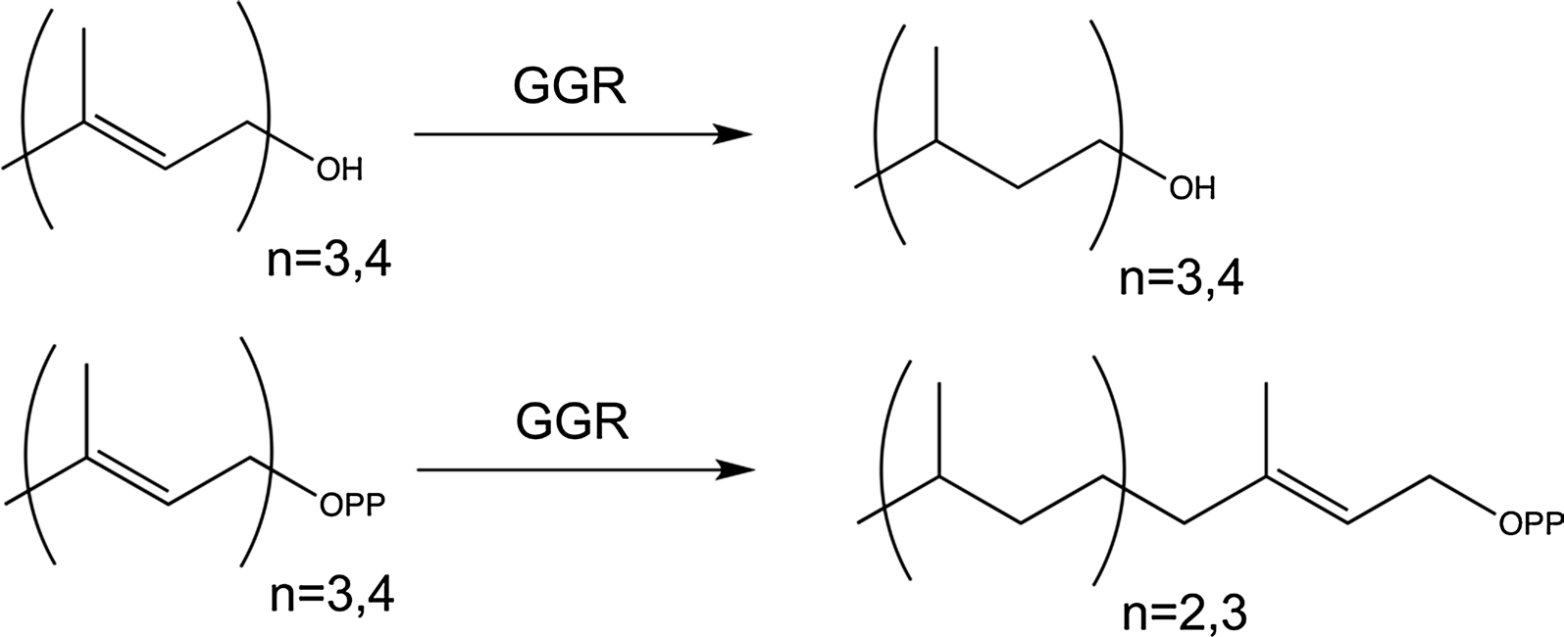
Products formed from prenyl alcohols (top) or pyrophosphates (bottom) when incubated with GGR

Biomanufacturing of reduced isoprenoid compounds requires a reductase activity under biologically relevant conditions required by bacterial and yeast strains (i.e., at 30–37 °C, at pH 7). In this study, we sought to increase the diversity space of GGRs by testing several dozen putative GGR sequences across a broad phylogeny, and we proceeded to test their associated substrate promiscuities under conditions ideal for microbial manufacturing (Scheme 1). Herein, we present significant insights on GGR activities that encompass newly confirmed GGR enzymes, novel substrate activities, and promiscuous catalysis.

## Results and discussion

### Selection and expression of potential GGR candidates

**Fig. 1:**
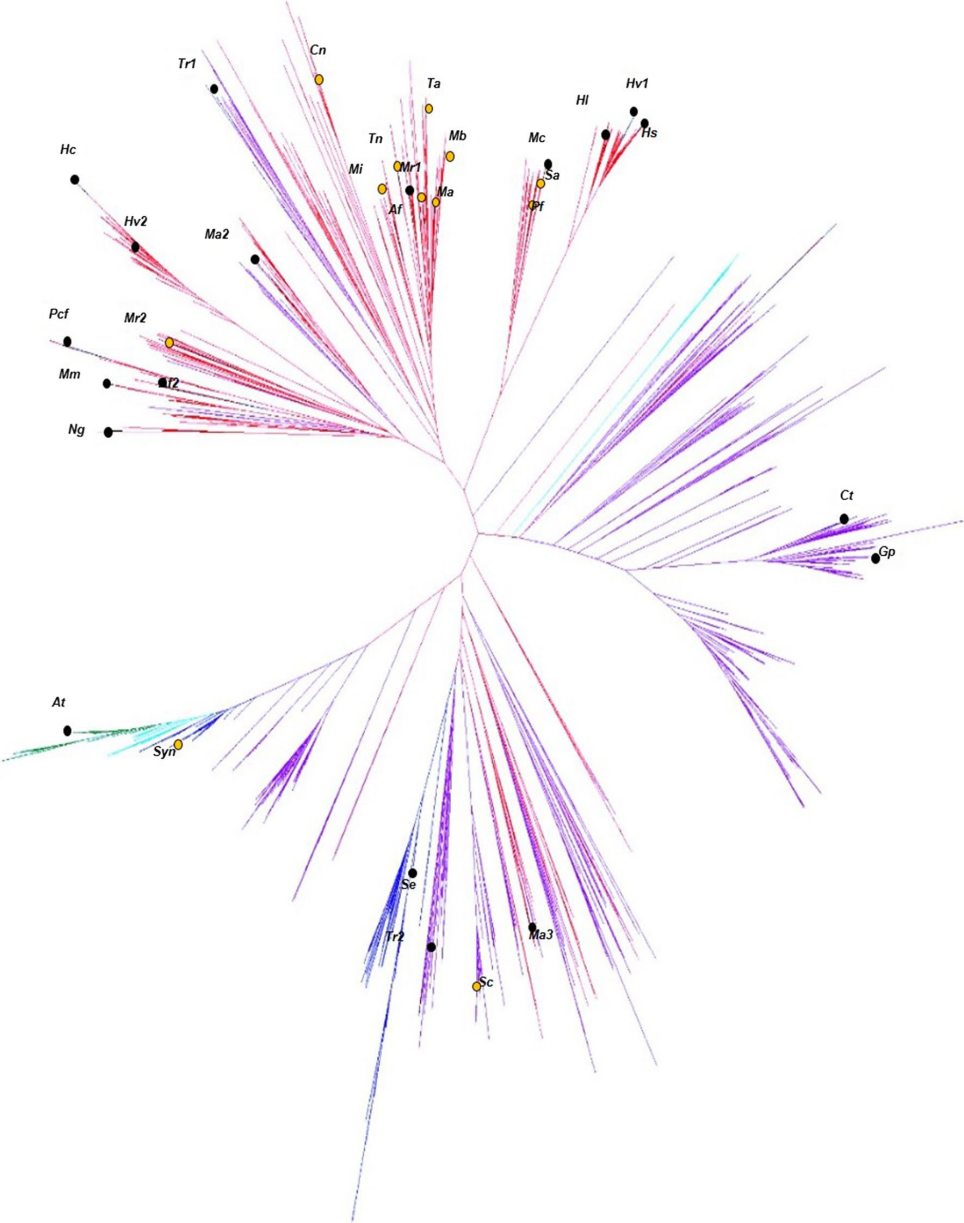
Phylogenetic tree representing the diversity of the GGR family of enzymes. The tree includes 1787 sequences of predicted GGRs from the InterPro database. Putative GGRs were selected from various organisms encompassing archaea (red), bacteria (purple), cyanobacteria (blue), alga (cyan), and plants (green). Black circles represent GGRs selected to test for isoprenoid reductase activity within this study; orange circles represent soluble proteins successfully purified and tested for reductase activity. The organismal abbreviations are listed described in Table 1

The Interpro database (EMBL-EBI) predicts over 8000 proteins present within the GGR family (InterPro code: IPR011777), with many homologous genes containing sequence identities as low as 20–30%. After sequence alignment, a phylogeny tree includes 1787 sequences of predicted GGR from the InterPro database. A few GGRs within this database have been confirmed by other groups to reduce a wide variety of large prenylated substrates, including GGPP, DGGGP, geranylgeranylchlorophyll, menaquinone, and dolichol [25–32]. To investigate the in vitro prenyl reductase potential of other genes within the GGR family, we selected some with conserved sequence homologies to known GGRs and other more distant sequences. As observed in Fig. 1, it was possible to observe some subgroups with conserved sequences (e.g., Mc, Sa, Pf or Hl, Hv1, Hs). Most of the sequences in the predicted GGR family, however, are very divergent. Our selection was then based on kingdom and diversity of species (e.g., archaea, algae, plant, cyanobacteria, and bacteria), on environmental diversity (e.g., temperatures, pH, aerobic or anaerobic), as well as particular characteristics of some strains (e.g., *Corynebacterium terpenotabidum* or *Gordonia polyisoprenivorans* are actinomycetes capable of degrading squalene and rubber reciprocally). A few GGRs were also selected more randomly for their atypical sequences (Fig. 1).

**Table 1:** Table of proteins tested for potential enzymatic isoprenoid reductase activity

GGR name	Organism	Type	INTERPRO number	Molecular weight (kDa)
AfGGR	*Archaeoglobus fulgidus #1*	Archaea	>A0A075WA57	44
Af2GGR	*Archaeoglobus fulgidus #2*	Archaea	>A0A075WDX8	42
AtGGR	*Arabidopsis thaliana*	Plant	>Q9CA67	49
CnGGR	*Candidatus Nitrosopumilus*	Archaea	>K0BBV2	47
CtGGR	*Corynebacterium terpenotabidum*	Bacteria	>S4XGC5	49
GpGGR	*Gordonia polyisoprenivorans*	Bacteria	>H6N2C4	46
HcGGR	*Halorubrum californiensis*	Archaea	>M0EA67	41
HlGGR	*Halostagnicola larsenii X*	Archaea	>W0JLI3	52
HsGGR	*Haloterrigena salina*	Archaea	>M0BU08	53
Hv1GGR	*Haloferax volcanii #1*	Archaea	>D4GXW9	53
Hv2GGR	*Haloferax volcanii #2*	Archaea	>D4H022	41
Ma1GGR	*Methanosarcina acetivorans #1*	Archaea	>Q8TQQ6	46
Ma2GGR	*Methanosarcina acetivorans #2*	Archaea	>Q8TLY0	47
Ma3GGR	*Methanosarcina acetivorans #3*	Archaea	>Q8TSV3	45
MbGGR	*Methanococcoides burtonii*	Archaea	>Q12WF0	46
McGGR	*Metallosphaera cuprina*	Archaea	>F4FYK4	53
MiGGR	*Methanocaldococcus infernus*	Archaea	>D5VQY0	45
MmGGR	*Methanococcus maripaludis*	Archaea	>Q6LXX0	45
Mr1GGR	*Methanobrevibacter ruminantium #1*	Archaea	>D3E3T0	45
Mr2GGR	*Methanobrevibacter ruminantium #2*	Archaea	>D3E430	51
NgGGR	*Nitrososphaera gargensis*	Archaea	>K0IKB9	43
PcfGGR	*Pyrococcus furiosus*	Archaea	>Q8U3L2	43
PfGGR	*Pyrolobus fumarii*	Archaea	>G0EHJ8	53
SaGGR	*Sulfolobus acidocaldarius*	Archaea	>M1I414	52
ScGGR	*Streptomyces coelicolor #1*	Bacteria	>Q9K426	47
SeGGR	*Synechococcus elongatus #1*	Cyanobacteria	>Q31QX9	43
SynGGR	*Synechocystis species*	Cyanobacteria	>L8ATV2	47
TaGGR	*Thermoplasma acidophilum #1*	Archaea	>Q9HKS9	45
TnGGR	*Thermococcus nautili*	Archaea	>W8NRH6	46
Tr1GGR	*Thermocrinis ruber #1*	Bacteria	>W0DGJ3	41
Tr2GGR	*Thermocrinis ruber #2*	Bacteria	>W0DID8	42

The molecular weight of the enzymes includes the N-terminal His tag sequence

**Fig. 2:**
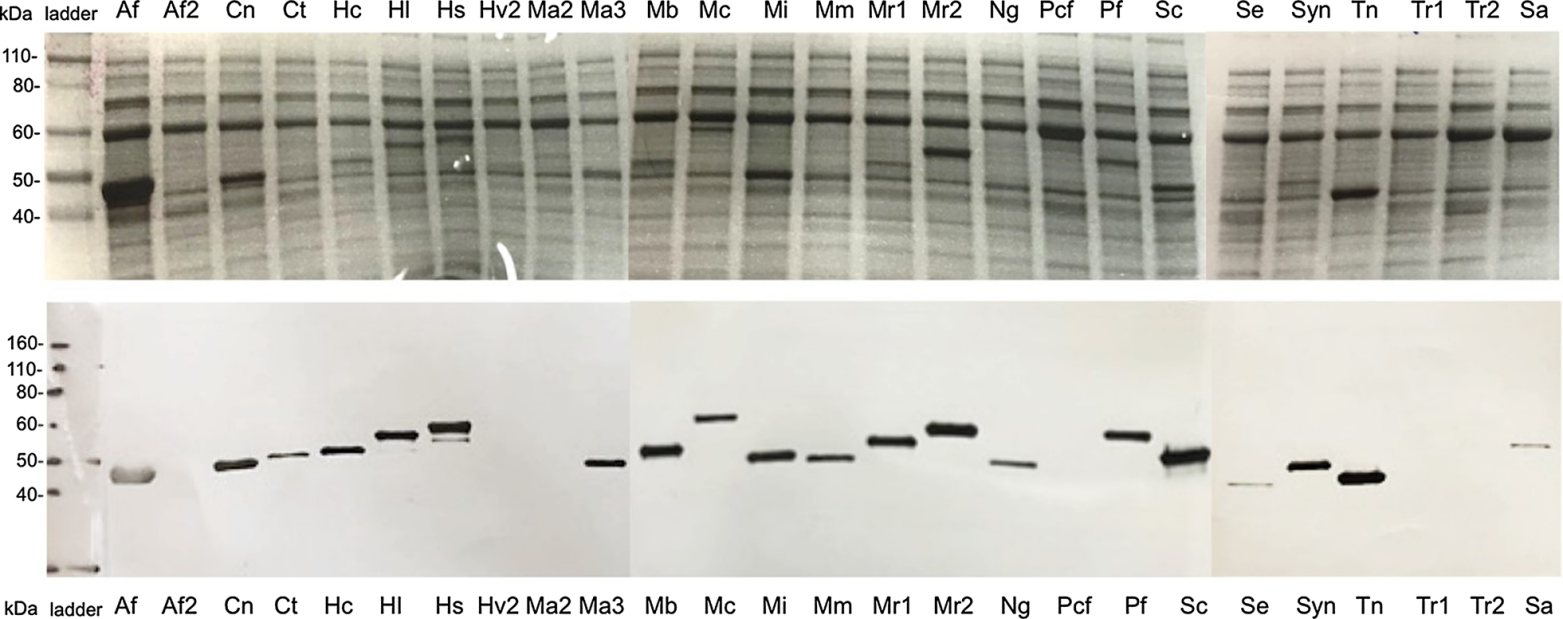
Various putative GGR’s expression in *E. coli* BL21 (DE3) harboring the pG-KJE8 plasmid. SDS-PAGE (top) and Western blot using anti-His antibody (bottom) verify protein overexpression in crude lysates at the expected masses. Only 26 out of the 31 selected GGR are shown in this figure

The 31 selected genes were codon optimized for *E. coli* expression and were all successfully transformed into *E. coli*. Initial expression attempts were not successful for many proteins using *E. coli* BL21 (DE3). However, by *E. coli* BL21 (DE3) strain harboring the commercially available pG-KJE8 plasmid overexpressing several *E. coli* chaperones, 24 of 31 strains overexpressed soluble proteins at the target masses for each protein, with each protein’s presence in cell lysates confirmed by western blot containing the anti-His tag antibody (Fig. 2). Out of them, only 12 proteins (Af, Cn, Ma, Mb, Mi, Mr2, Pf, Sa, Sc, Syn, Ta, and Tn) were obtained in sufficiently large quantities needed for activity assays after a standard purification and concentration process conducted at pH 7.4.

### In vitro activity with isoprenoid alcohols

The 12 soluble proteins successfully isolated were tested for reductase activity on GGOH and FOH, and products obtained after enzymatic incubation were analyzed by GC–MS.

**Fig. 3:**
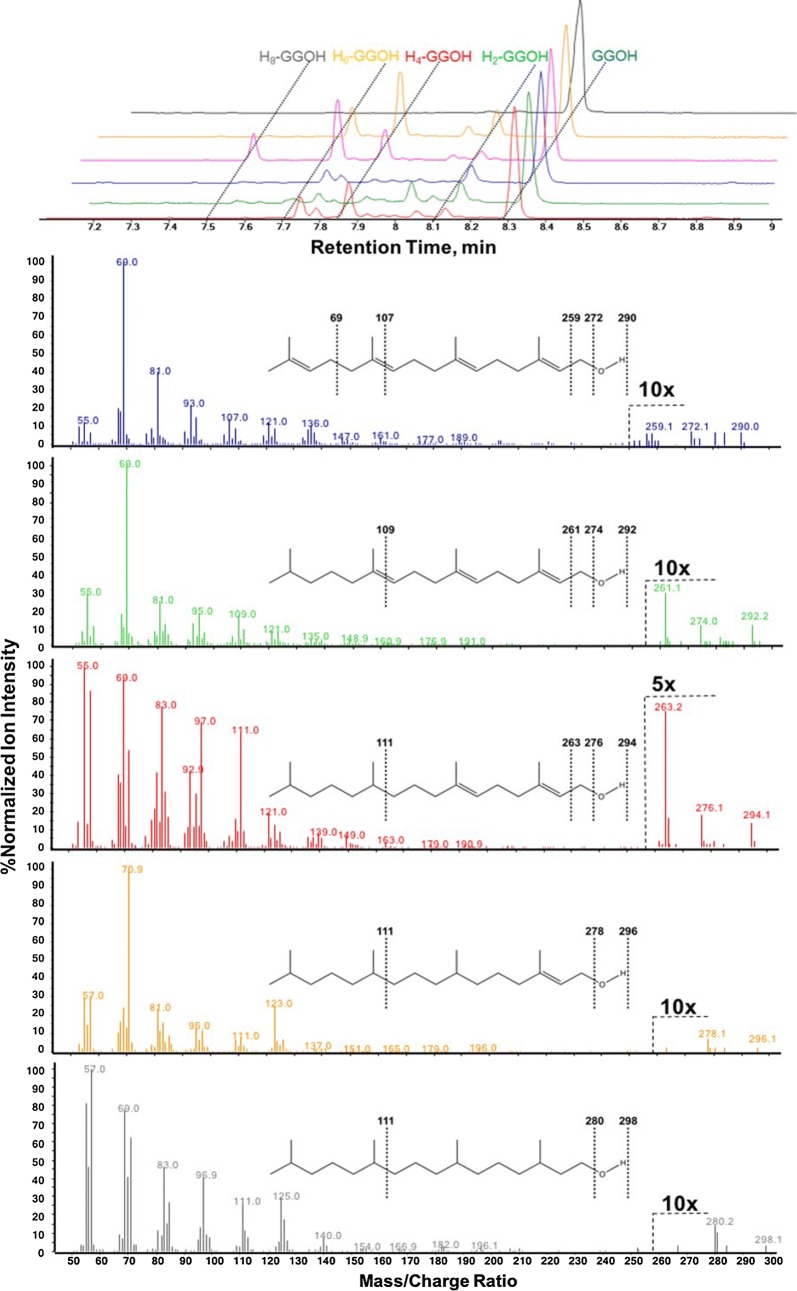
(Top) normalized TIC profiles of five putative GGRs (Af, red; Pf, green; Mi, blue; Sa, magenta; Tn, orange; no enzyme, black) found to reduce GGOH (RT = 8.31 min) to H_2_-GGOH (RT = 8.12 min), H_4_-GGOH (RT = 7.85 min), H_6_-GGOH (RT = 7.72 min), and H_8_-GGOH (RT = 7.52 min) upon 1 h incubation under standard assay conditions. All peaks elute with a relative error of ± 0.05 min. (Bottom) the associated mass spectra for GGOH (blue), H_2_-GGOH (green), H_4_-GGOH (red), H_6_-GGOH (orange), H_8_-GGOH (gray) are shown with signature ions used for structural assignment of products

**Fig. 4:**
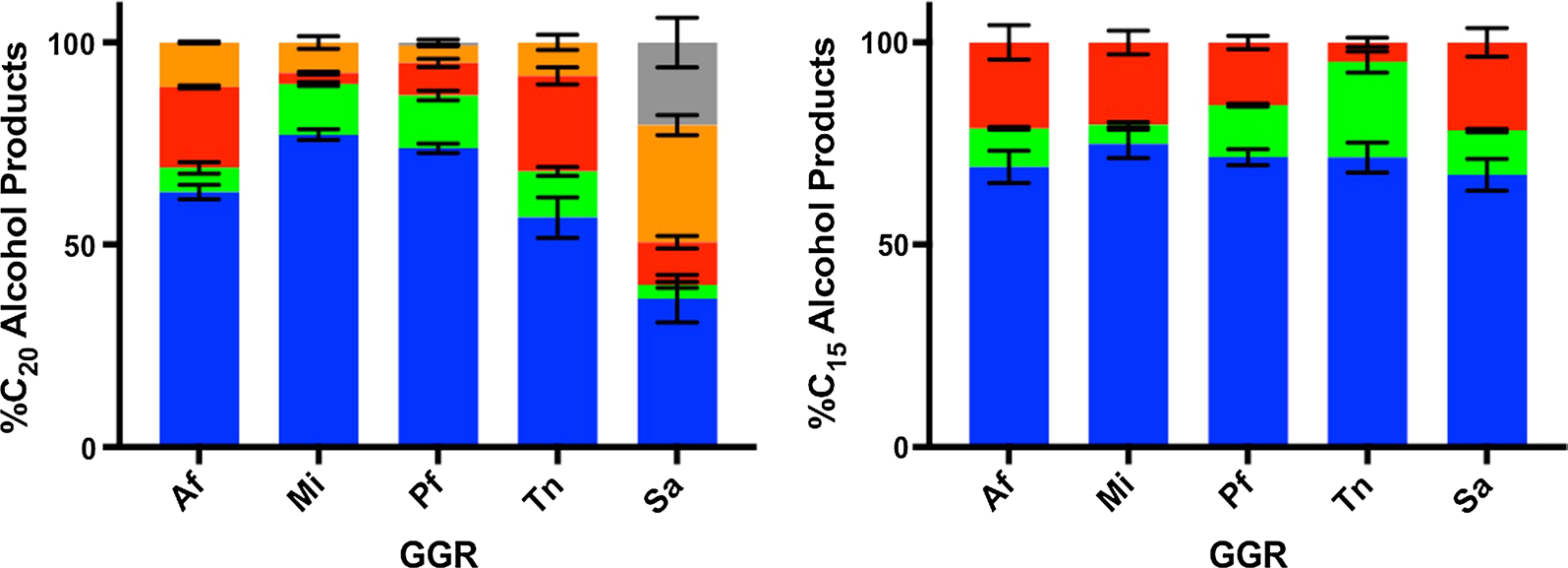
Endpoint activity profiles for GGR reduction of either GGOH (left) or FOH (right) incubated under standard assay conditions for 1 h. Product distributions are represented as relative percentages of unreduced substrate (blue), H_2_-GGOH or H_2_-FOH (green), H_4_-GGOH or H_4_-FOH (red), H_6_-GGOH or H_6_-FOH (orange), and H_8_-GGOH (gray)

Out of the 12 purified GGRs, five were discovered to enzymatically reduce geranylgeraniol (GGOH). Neat GGOH substrate eluted at a retention time (RT) of 8.4 ± 0.1 min (Fig. 3), with a directly proportional TIC response ranging from 0 to 200 µM (Additional file 1: Figure S1). Upon incubation with any of five putative GGRs isolated from *Archaeoglobus fulgidus* (AfGGR), *Methanocaldococcus infernus* (MiGGR), *Pyrolobus fumarii* (PfGGR), *Thermococcus nautili* (TnGGR), or *Sulfolobus acidocaldarius* (SaGGR), several peaks eluting earlier than 8.4 min were observed (Fig. 3). These peaks were assigned to structures of H_2_-GGOH (RT = 8.1 ± 0.1 min), H_4_-GGOH (RT = 7.9 ± 0.1 min), and H_6_-GGOH (RT = 7.7 ± 0.1 min). Moreover, as protein concentration was increased, substrate consumption accelerated (Additional file 1: Figure S2) with a concomitant increase in the formation of the various product peaks (data not showed), confirming enhanced isoprenoid reduction in the presence of higher concentration of enzymes. Out of all enzymes tested, SaGGR was the most active toward GGOH, with a specific activity of at least 50 ± 10 nmol terpenoids reduced per milligram of enzyme per hour (Fig. 4 and Table 2). Typically, 70% of the initial GGOH would be recovered regardless of the varying amounts of reduced product formed. Hence, we assumed that all unrecovered substrate was unreduced, and the turnover numbers presented herein most likely represent a lower bound for reductase activity.

The H_2_-GGOH and H_4_-GGOH peaks have respective prevalent ion abundances at 261 and 263 *m*/*z*, which can be achieved by loss of a 31 Da [M–CH_2_OH] fragment during ionization and subsequent formation of a resonance-stabilized singly or doubly reduced geranylgeranyl fragment. Such fragments most likely originate from the prenyl units distal from the alcohol group being reduced first, in accordance with previous mechanistic proposals performed using various substrates on a variety of GGRs [29, 32, 33]. Moreover, the H_6_-GGOH peak matches with a phytol peak from the NIST database with > 90% probability, further reinforcing a mechanism of serial reduction of substrate beginning with the *δ*-prenyl group. Interestingly, several GGRs exhibit unknown side-products, with the most prevalent behavior observed between the H_2_-GGOH and H_4_-GGOH peaks in *Pyrolobus fumarii* GGR (RT = 8.0 min) (Fig. 3). This peak contains aberrant patterns for prenyl units within *m/z* window of 50–100, and we suggest these are H_4_-GGOH regioisomers in which one or both internal prenyl units are reduced first, which was suggested from the NIST database with > 80% probability (Additional file 1: Figure S3).

**Fig. 5:**
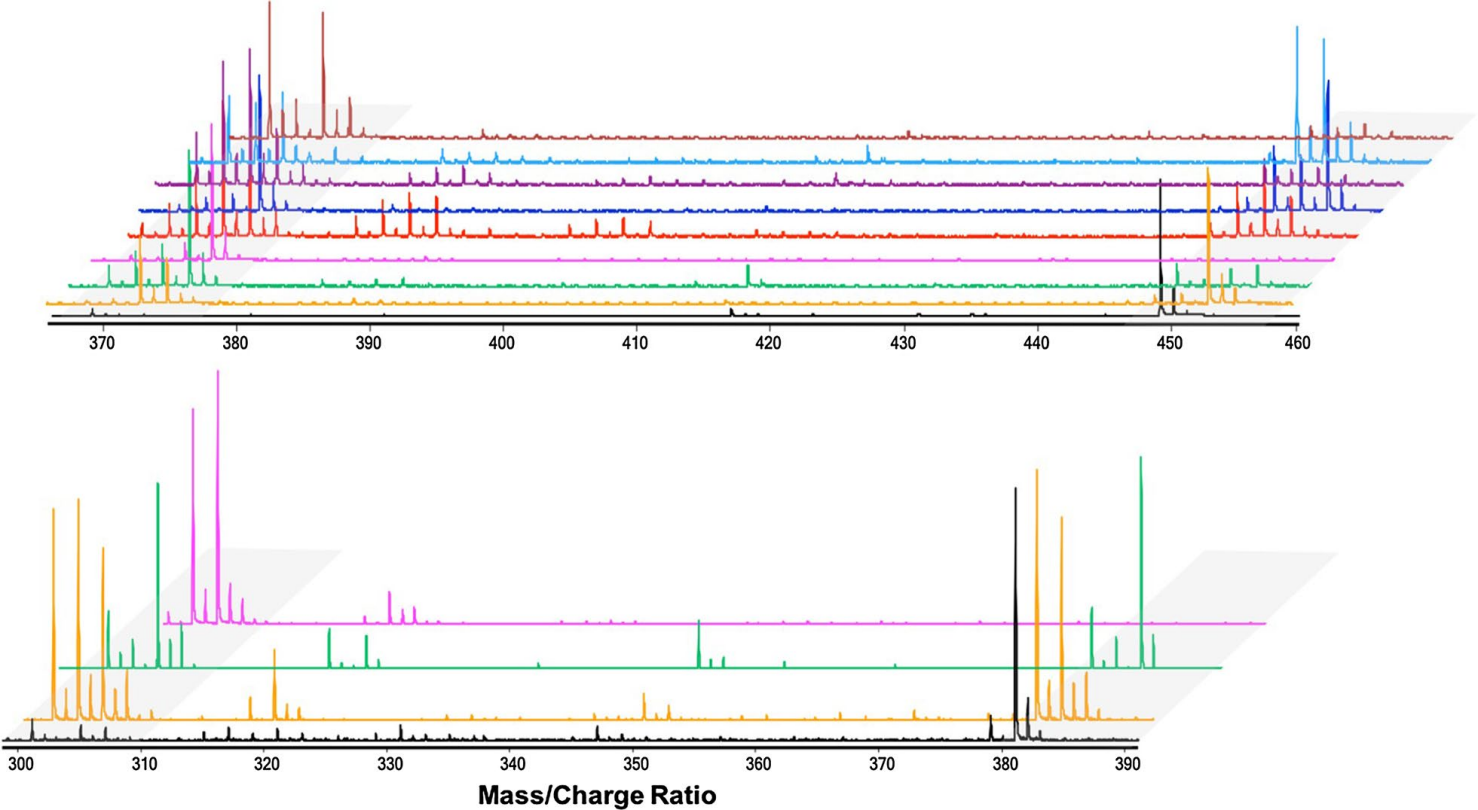
(Top) normalized MS–TOF spectra for eight putative GGRs (no enzyme, black; Tn, orange; Pf, green; Sa, magenta; Af, red; Mi, blue; Sc, purple; Ta, cyan; Ma, brown) found to reduce GGPP (*m*/*z* 449–457, highlighted in gray) or GGP (*m*/*z* 369–377, highlighted in gray). (Bottom) normalized MS–TOF spectra for the three putative GGRs (Tn, orange; Pf, green; Sa, magenta) found to reduce FPP (*m*/*z* 381–387, highlighted in gray) or FP (*m*/*z* 301–307, highlighted in gray). Reduced products are signified by abundances present at increases of ca. 2 Da from GGPP, GGP, FPP, or FP

Of most noteworthy interest is the product eluted at 7.5 ± 0.1 min RT from assays containing GGRs from *Sulfolobus acidocaldarius* (Fig. 3). The mass spectra are matched against the 3,7,11,15-teramethylhexadecan-1-ol compound, a complete hydrogenation product of GGOH, in the NIST database with > 88% probability. SaGGR, among others, has been demonstrated to reduce 3 out of 4 prenyl units of GGPP at best as observed in this work and others [29, 33]. Because a complete reduction is not observed in isoprenoid pyrophosphate substrates (Fig. 5) but is observed in the isoprenoid alcohol (Fig. 3), it seems that the absence of phosphate groups might facilitate enhanced diffusion of the α-prenyl group to the flavin reducing site in the alcohol substrates, leading to a fully reduced product. To our knowledge, this is the first evidence of any nonnative isoprenoid substrate undergoing full reduction by any known or putative GGR enzyme.

**Fig. 6:**
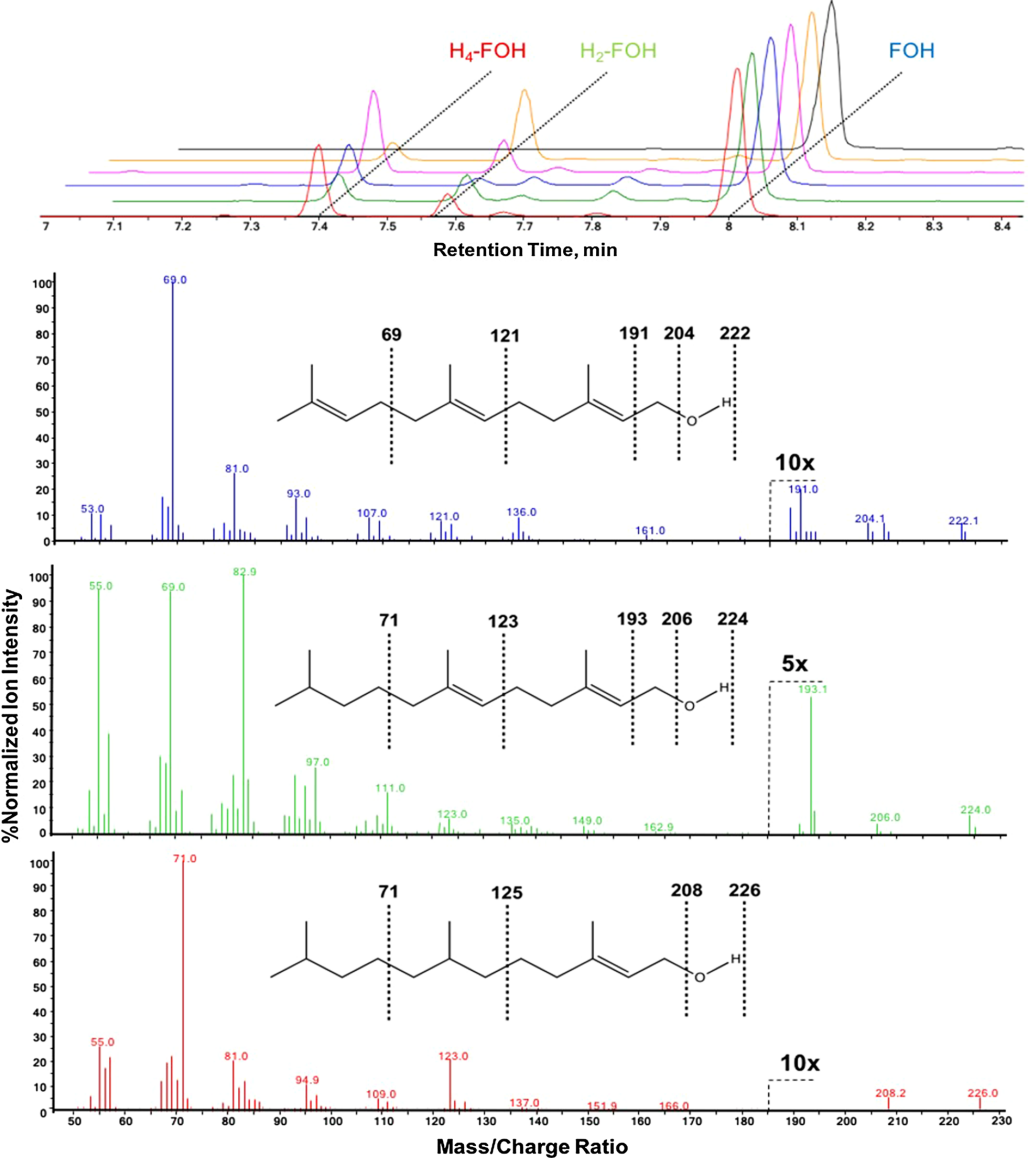
(Top) normalized TIC profiles of five putative GGRs (Af, red; Mi, green; Pf, blue; Sa, magenta; Tn, orange; no enzyme, black) found to reduce FOH (RT = 8.00 min) to H_2_-FOH (RT = 7.58 min) or H_4_-FOH (RT = 7.40 min) upon 1 h incubation under standard assay conditions. All peaks elute with a retention time error of ± 0.05 min. (Bottom) the associated mass spectra for each peak are shown with signature molecular ions for structural assignment of products

Similarities in reducing activity were also prevalent using farnesol as a substrate. The unreduced FOH substrate eluted with a RT of 8.0 ± 0.1 min, with the putative singly (H_2_-FOH) and doubly reduced (H_4_-FOH) farnesol eluting at 7.6 ± 0.1 min and 7.4 ± 0.1 min, respectively (Fig. 6). Farnesol ionization was also directly proportional to concentration ranging from 0 to 200 µM (Additional file 1: Figure S1). The accompanying mass spectrum for H_2_-FOH reveals a similar ionization pattern to that observed in H_2_-GGOH via the prevalence of a strong 193 *m/z* peak. This parallels the H_2_-GGOH peak pattern containing one less prenyl group (*m/z* = 70 Da). This suggests that the terminal isoprenoid unit is also reduced first in farnesol, conserving the enzymatic reduction mechanism regardless of substrate. The H_4_-FOH peak at 7.4 min more closely resembles the H_6_-GGOH peak, with identical peak groupings near the 71, 81, and 123 *m/z* parent fragments.

Unlike GGOH, all GGRs appeared to have similar levels of FOH products under standard assay conditions, exhibiting an average specific activity of 7 ± 2 nmol terpenoid groups reduced mg^−1^ enzyme h^−1^ (Fig. 4 and Table 1). Notably, reduction patterns in TnGGR on FOH differ slightly from the other GGRs under standard assay conditions, as its major product is H_2_-FOH instead of H_4_-FOH (Fig. 4). No fully reduced farnesol peaks were observed under standard assay conditions nor at enzyme concentrations as high as 150 µM at pH 7.4. However, SaGGR generated a modest amount of H_6_-FOH when incubated under the enzyme’s optimal conditions at 50 °C and pH 5.5 [33] (Additional file 1: Figure S4).

Compared to GGOH, emergent side products are less prevalent in the farnesol TICs. Whereas multiple peaks were observed between the singly and doubly reduced GGOH (Fig. 3), a single prevalent peak at 7.8 min elutes between FOH and H_2_-FOH, mainly observed when incubated in the presence of Pf and MiGGR (Fig. 6). The associated mass spectrum is tentatively assigned to a regioisomer of H_2_-FOH where the middle prenyl unit is reduced first (Additional file 1: Figure S5). The spectrum matches the NIST database for (*E*)-3,7,11-trimethyldodeca-2,10-dien-1-ol with a probability of 85% (Additional file 1: Figure S5). Many of the aberrant mass groupings between 50 and 100 *m/z* (Additional file 1: Figure S5) parallel those observed in the GGOH reaction incubated with PfGGR (Additional file 1: Figure S3). However, the 7.8-min peak does not contain the *m/z* 193 ion. This ion could be formed by cyclization of a [M–CH_2_OH] fragment containing a reduced terminal prenyl group and is absent in products where the middle group is reduced first due structural rigidity associated with the remnant α- and γ-prenyl groups. This observation, coupled with the aberrant TIC product profile observed with GGOH, suggests a promiscuous mechanism in which PfGGR has been observed to reduce prenyl monomers out of order with respect to their polymeric structural order.

### In vitro activity with isoprenoid pyrophosphates

The 12 soluble GGRs successfully purified were tested for reductase activity on FPP and GGPP, and products were detected by LC–MS–TOF. Both farnesyl pyrophosphate (FPP, *m/z* = 381.123 ± 0.001 Da) and geranylgeranyl pyrophosphate (GGPP, *m/z* = 449.183 ± 0.002 Da) standards eluted with a retention time of 1.70 ± 0.05 min (Fig. 5); both substrates produced linear standard curves over a concentration range of 0–120 µM (Additional file 1: Figure S6). When incubated with GGR under standard assay conditions, reduced isoprenoid products were observed to co-elute with fully oxidized substrate under isocratic LC conditions. Therefore, only normalized LC–MS–TOF spectra were utilized to distinguish the relative levels reduced and oxidized compounds that co-elute after incubating with GGRs isolated from various species (Additional file 1: Table S1).

Interestingly, all proteins in this study discovered to enzymatically reduce prenyl pyrophosphates revealed co-eluting side products indicative of substrate or product hydrolysis of one phosphate moiety (Fig. 5). Increased abundances of farnesyl monophosphate (FP, *m/z* = 301.177 ± 0.001 Da) or geranylgeranyl monophosphate (GGP, *m/z* = 369.213 ± 0.003 Da) only emerged when incubated with enzyme; minimal hydrolysis was observed in GGPP or FPP standards and relative GGPP/GGP and FPP/FP ratios remained constant as a function of time in negative controls ran without enzyme (Additional file 1: Figure S7). Structural studies of SaGGR crystallized with GGPP revealed three distinct substrate binding modes with varying degrees of phosphorylation within each binding position [33]. Within the catalytically relevant binding mode, both phosphate moieties are resolved. In the other two binding modes, however, either one or zero phosphate group was structurally resolved. This was attributed to dephosphorylation during the crystallization process [33]. Herein, we observed a time-dependent emergence of hydrolyzed monophosphate products via LC–MS–TOF; yet it still requires further characterization how the enzyme facilitates this phenomenon while conducting substrate reduction.

**Fig. 7:**
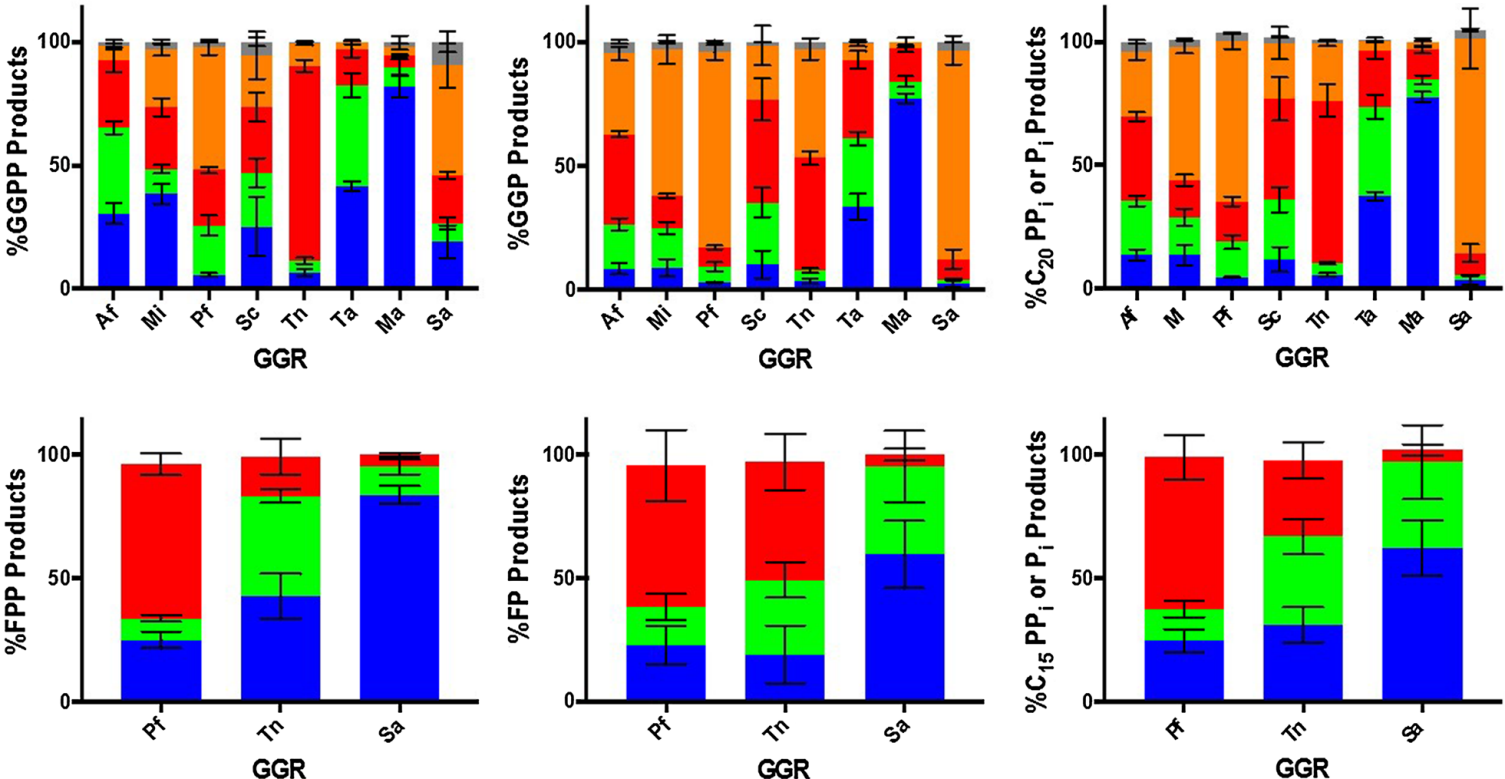
Endpoint activity profiles for GGR reduction of either GGPP (top) or FPP (bottom) incubated under standard assay conditions for 1 h. Product distributions are represented as relative percentages of unreduced substrate (blue), H_2_-products (green), H_4_-products (red), H_6_-products (orange), and H_8_-products (gray) for intact isoprenoid pyrophosphates (left column), hydrolyzed monophosphates (middle column), and the total intensity (right column)

Reductase activity on FPP and GGPP varied from what was observed on alcohol substrates (Fig. 7). Indeed, none of the GGRs tested could significantly reduce all vinyl groups within FPP or GGPP even when GGRs were incubated under the optimal condition for enzyme activity (at 50 °C and pH 5.5) (data not shown). Out of the five GGRs found to reduce FOH, only PfGGR, TnGGR, and SaGGR could reduce FPP. On the other hand, three GGRs isolated from *Streptomyces coelicolor* (ScGGR), *Methanosarcina acetivorans* (MaGGR), and *Thermoplasma acidophilum* (TaGGR) were found to reduce GGPP along with the five GGRs demonstrating reductase activity toward GGOH. Most GGRs that have been isolated thus far were from archaea; to our knowledge, ScGGR is the first bacterial GGR demonstrated to reduce GGPP. Due to unexpected hydrolysis of one phosphate moiety under standard assay conditions, specific activities for reduction were not quantified for any GGR. However, relative reductase activities can be gleaned by quantifying the proportion of reduced and unreduced compounds present within intact or hydrolyzed mass groupings (Fig. 7). For example, the relative ion intensities of each singly reduced product (H_2_-FPP or H_2_-FP) present is normalized to the sum of FPP, H_2_-FPP, H_4_-FPP, H_6_-FPP, FP, H_2_-FP, H_4_-FP, and H_6_-FP extracted ion intensities.

Interestingly, all GGRs revealed a larger proportion of reduced products present as hydrolyzed moieties than non-hydrolyzed moieties (Fig. 7). To discern a correlation between enzymatic phosphate hydrolysis and enzymatic reduction of prenyl monophosphates, we assayed PfGGR and SaGGR as a function of time, as these enzymes are representative of low and high amounts of monophosphate found after standard assay incubation, respectively (Additional file 1: Figure S7). Indeed, substrate hydrolysis appears to react as a first-order exponential process which occurs more quickly in SaGGR than for PfGGR (Additional file 1: Figure S8). Moreover, the relative abundances of reduced monophosphate products increase over time in both assays, indicating that most GGRs can still reduce FP or GGP as a substrate during substrate hydrolysis (Additional file 1: Figure S9). Such an inference is reasonable considering many GGRs assayed enzymatically reduce terpenoid alcohols and pyrophosphates.

Promiscuous hydrolysis complicates any interpretations regarding which enzymes are most active toward a given substrate due to the inability to quantify the MS response of terpenoid phosphates. However, it can be inferred that all GGRs can reduce between 5 and 10 nmol prenyl groups of FPP or GGPP mg^−1^ enzyme h^−1^. The turnover number would be modestly elevated for GGPP reduction, as all C_20_ species are extracted as some partially reduced product within error after 1 h. Such turnover numbers are in line with other reports on GGRs with a variety of substrates [32, 33].

### Structural insights and mechanistic implications

Several synthetic approaches are currently being explored to perform selective hydrogenation on a few substrates [34–36]. Biological systems such as enoyl-CoA reductase and old yellow enzyme exhibit a similar oxidoreductase activity to GGR, yet benefit from active sites that enhance the electron-withdrawing nature of α,β-unsaturated carbonyl substrates [17–20]. Patented ene-reductases utilizing old yellow enzyme as a scaffold enhance reductase activity on a variety of substrates by evolving active sites complementary to a variety of electron withdrawing groups among a diverse variety of α,β-unsaturated substrates [37]. However, an evolved GGR active site designed for isoprenoid reduction would probably require significant divergence from these scaffolds since they do not utilize electron-withdrawing activation for alkene reduction [30].

Of the eight proteins that were identified as GGRs active toward terpenoid alcohols and/or terpenoid pyrophosphates, five (Sa-, Pf-, Af-, Mi-, and TnGGRs) were isolated from archaeal organisms that optimally thrive under hyperthermophilic conditions (i.e., *T* ≥ 80 °C). SaGGR, TaGGR, and AfGGR have been identified to reduce various large intermediates (i.e., larger than 20 carbons) associated with archaeal lipid biosynthesis, with GGPP or GGOH serving as the smallest substrates known to undergo prenyl reduction [27, 29, 32]. In this study, we have significantly expanded the known GGR substrate activity profiles, demonstrating multiple prenyl group reduction in GGOH and FOH within all five hyperthermophilic GGRs.

In addition to the five GGRs active on alcohols, TaGGR, MaGGR, and ScGGR also sufficiently reduced GGPP or GGP (Figs. 5 and 7). However, only PfGGR, SaGGR, and TnGGR were found to reduce the smaller FPP or FP substrates. Because the relative amount of H_2_-, H_4_-, and H_6_-GGOH increase in relative abundances within the monophosphate mass groupings relative to the pyrophosphate mass groupings, it can be inferred that prenyl monophosphates are also substrates reduced by several GGRs (Additional file 1: Figure S9). This seems suitable given the ability of several GGRs to reduce prenyl alcohols.

**Fig. 8:**
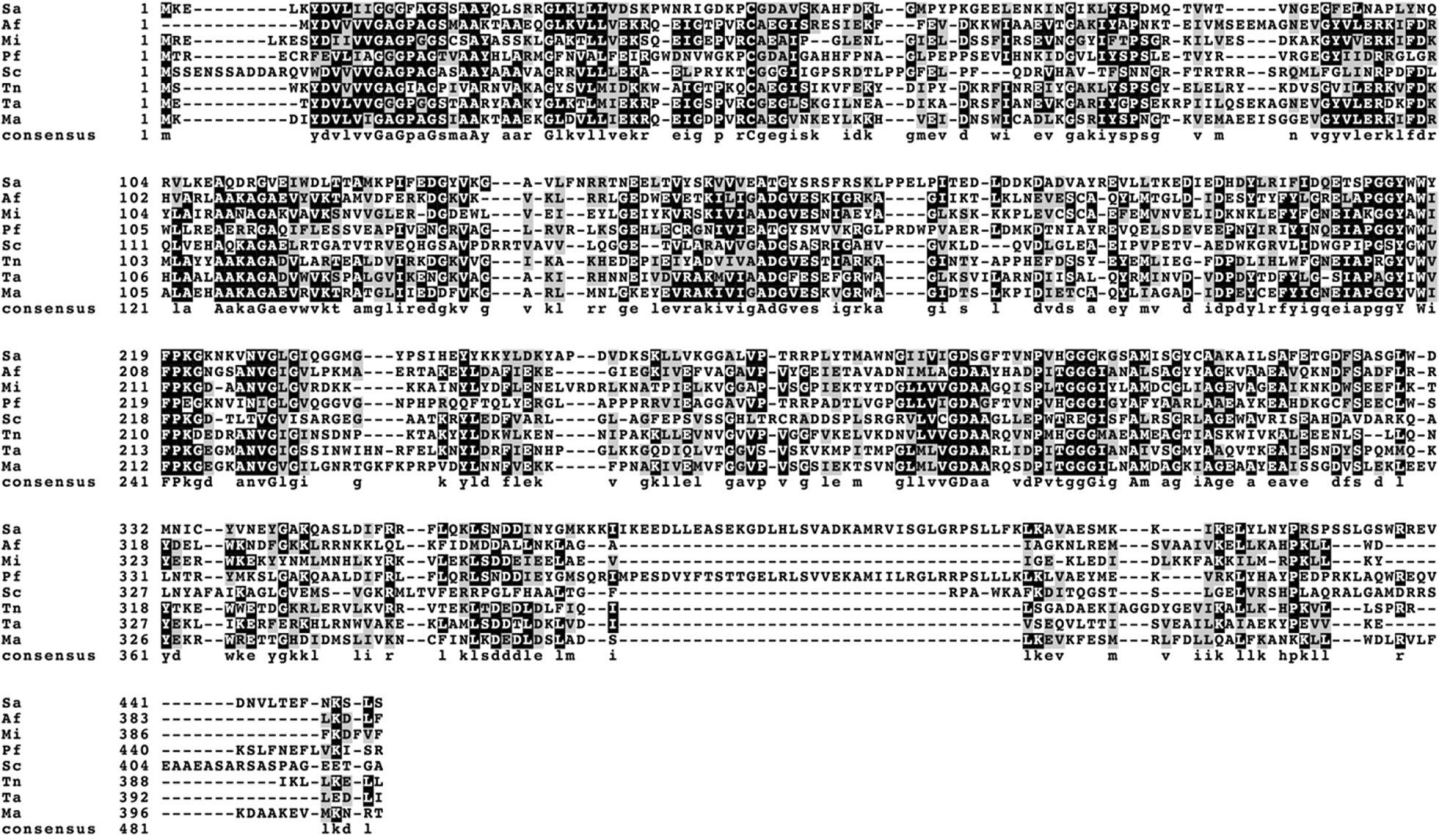
Multiple sequence alignment of proteins shown to enzymatically reduce either C_15_ or C_20_ prenyl alcohols or prenyl pyrophosphates. Identical residues are highlighted in black, similar residues are highlighted in grey, and gaps are represented by dashes

A structural alignment of all eight active GGRs reveals very little commonalities among all protein sequences with known crystal structures: SaGGR and TaGGR, with PfGGR ca. 46% identical to SaGGR and MaGGR, MiGGR, and AfGGR ca. 40–46% identical to TaGGR (Fig. 8). SaGGR and TaGGR contain three domains: an FAD binding domain, a catalytic domain, and a C-terminal domain [29, 30]. While sequence identities remain low among all demonstrably active GGRs, certain key structural motifs remain conserved within their predicted FAD binding domains and catalytic domains. Of the two known crystal structures of active GGRs, both contain an active site cysteine (Cys47 in SaGGR; Cys45 in TaGGR) thought to serve as a critical redox modulator within the active site during reduction. All GGRs shown to reduce either isoprenoid alcohols or pyrophosphates contain this critical cysteine within their cofactor binding domains, suggestive of a conserved electron transfer mechanism. In addition, all sequences predicted catalytic domains contain the YXWXFP (SaGGR residues 215–220) and GGG motifs (SaGGR residues 298–300) believed to modulate substrate interactions and assist in substrate diffusion through the reduction center.

**Fig. 9:**
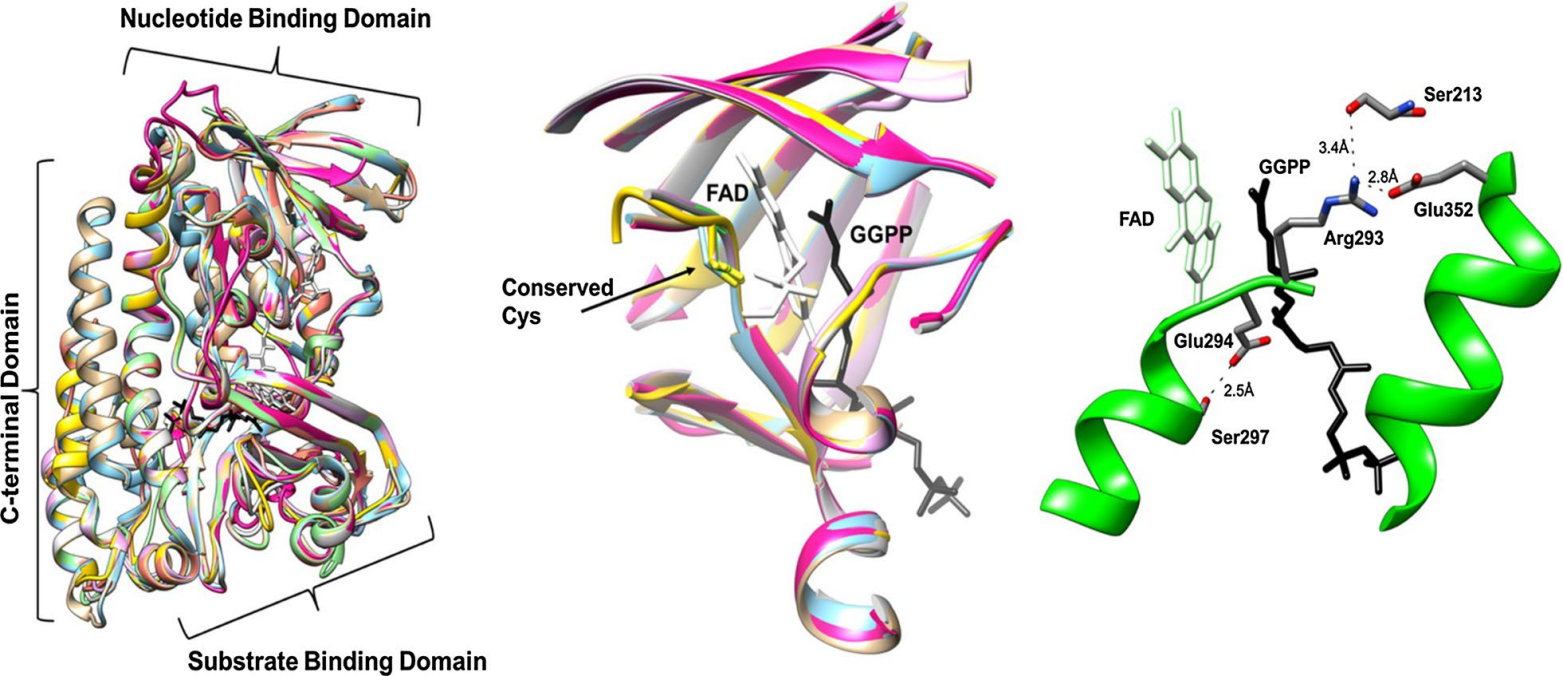
(Left) overlaid prediction of modeled protein structures of proteins (Sa, cyan; Pf, tan; Af, fuschia; Mi, green; Tn, red; Ta, gray; Sc, magenta; Ma, orange) with demonstrated GGR activity using SaGGR (PDB: 4opd) as a template. (Middle) overlaid alignment of protein active sites of residues within 10 Å of either the FAD isoalloxazine ring or GGPP substrate. The conservation of the active site cysteine found in all GGRs (cf. Fig. 8) are found in proper position to modulate the redox properties of the cofactor. (Right) examination of the ScGGR active site containing the divergent REG catalytic motif relative to the GGG motif found in archaeal GGRs. Arg293 and Glu294 of ScGGR make critical intradomain hydrogen bonding interactions to accommodate the GGPP binding site

Protein structures of aligned sequences were predicted using either SaGGR or TaGGR as a template. While there is a fair amount of expected structural divergence among the structures’ surfaces, a comparison of the active sites reveals a fair degree of similarity in topology (Fig. 9). However, some of the structural motifs strictly conserved among all archaeal GGRs exhibit significant divergence within ScGGR, the only known GGR to be isolated from a bacterial organism. While all archaeal GGRs studied in this work possess a YXWXFPX_7-8_GXG motif, the terminal glycine is mutated to isoleucine in ScGGR. Even more interestingly, the GGG motif has significantly diverged to REG in ScGGR. In several GGRs from photosynthetic organisms with demonstrated capability to reduce prenylated chlorophyll, *Rhodobacter sphaeroides*, *Synechocystis* sp. *PCC 6803*, and *Arabidopsis thaliana*, this motif was found to be GEG [26, 38, 39]. It seems that non-archaeal GGRs utilize preferentially charged residues within this critical catalytic region to either enhance polar interactions on prenylated substrates containing polar groups or to introduce critical hydrogen-bonding interactions that help maintain the integrity of the substrate tunnel during reduction (Fig. 9).

Mechanistic interpretations from other groups propose that the prenyl group closest to the pyrophosphate moiety (α-prenyl group) remains oxidized in GGPP and FPP. This observation additionally applies to their monophosphate counterparts in this work, FP and GGP. All enzymes tested to date seem to conserve this characteristic of avoiding reduction at the α-position on phosphate intermediates, aligning with current paradigms that auxiliary prenyl reductases are responsible for reducing this group in archaea and eukaryotes [40].

To our knowledge, full isoprenoid reduction by GGR has only been observed with its natural C_40_ isoprenoid substrate DGGGP. In this work, we observed full reduction for the first time on smaller (i.e., C_20_ or C_15_) isoprenoid alcohol substrates, namely GGOH and FOH with SaGGR (Figs. 3, 4, 6, Additional file 1: Figure S4). Interestingly, the absence of phosphate groups appears to assist in full substrate reduction. Analysis of the catalytically relevant binding mode of GGPP in SaGGR reveals that binding site residues His55 and Asn90 could provide hydrogen bonding interactions with phosphate moieties that could prevent the α-prenyl group from being reduced [33]. Alcohol substrates may not interact as strongly with these residues, facilitating a degree of full reduction unobserved in pyrophosphate substrates. Why some enzymes reduce isoprenoid alcohol and pyrophosphate substrates, while others only reduce isoprenoid pyrophosphates requires further structural characterization.

## Conclusions

In this study, we have significantly expanded the possible activities among proteins demonstrated to enzymatically reduce prenyl pyrophosphates or prenyl alcohols. We have demonstrated (1) the discovery of four novel protein sequences (PfGGR, MiGGR, ScGGR, and TnGGR) that have confirmed GGR activity in vitro in addition to expanded observed activities among previously characterized GGRs; (2) that several GGRs can reduce C_15_ terpenoid substrates, substrates smaller than reported substrates for GGR activity; (3) the complete reduction of double bonds on any C_20_ or C_15_ isoprenoid using SaGGR; (4) reductase activity on terpenoid monophosphates formed from hydrolysis of pyrophosphate substrates under reducing conditions in vitro; (5) the quantification of reductase specific activity on terpenoid alcohols; and (6) the confirmed isoprenoid reductase activity of the second known non-archaeal enzyme, as observed in the GGR isolated from *Streptomyces coelicolor*.

This demonstration of protein expression and reductase activity at neutral pH and low temperature highlights their potential suitability for integration into *S. cerevisiae* or *E. coli*. Moreover, the confirmation of reduction on C_15_ isoprenoids instantly expands the metabolic engineering potential for organisms producing sterol and squalene-derived isoprenoids. There are still unresolved issues to address for a direct application of these newly discovered GGRs to manufacture reduced isoprenoids. For example, more engineering will be needed on these enzymes to avoid enzymatic hydrolysis of isoprenoid pyrophosphates and to improve their activities especially at mesophilic condition. Nonetheless, this study demonstrated significant substrate promiscuity among these GGRs and could potentially open new pathways for isoprenoid-based polymers, chemicals, or biofuels by allowing for upstream reduction of various intermediates within the heavily utilized MEV or DXP terpene biosynthesis pathways.

## Methods

All chemicals and reagent were purchased from Sigma-Aldrich (St. Louis, MO), unless otherwise indicated. (*E*,*E*)-farnesol was purchased from Alfa Aesar (Haverhill, MA) and glycerol from VWR (Westchester, PA). Solvents for high performance liquid chromatography (HPLC) were purchased from HoneyWell Burdick and Jackson (Morristown, NJ) and were of HPLC grade or higher. Ammonium carbonate (30–33% NH_3_ basis) was purchased from Fluka Analytical Sigma-Aldrich (St. Louis, MO). Restriction enzymes and polymerases were purchased from New England Biolabs (Ipswich, MA).

### Sequence analysis and GGR homology

Multiple sequence alignments for potential GGR hits were generated using MUSCLE v. 3.8.31 and visualized using Geneious 7.0.6 [41, 42]. Sequences were curated manually, and phylogeny trees were computed using the maximum likelihood tree within the RAxML Software package, v. 8.1.24 under the LG plus gamma model of evolution (PROTGAMMALG in the RAxML model section) [43]. The MRE-based bootstrapping criterion was automatically determined for phylogeny tree construction. Annotation of the tree was performed in Itol [44]. After verification of GGR activity, the active enzymes underwent a second multiple sequence alignment and modeled for their predicted protein structures via SWISS-MODEL-PDB using either SaGGR or TaGGR as templates [45]. Active site geometries and local structures for all proteins were visualized using Chimera [46].

### Plasmid synthesis and transformation

The gene encoding SaGGR was amplified by PCR from the pSKB3-SaGGR plasmid using the forward (5′-GATATACATATGAAGGAACTTAAATATGACGTTCTG-3′) and reverse (5′-GTCGACGGAGCTCGAACTTAAACTTTTGTTAAACTCTGTTAGAAC-3′) primers synthesized by Integrated DNA Technologies [33]. The PCR fragment was digested at the *Nde*I and *Sac*I restriction sites and cloned into the pET-24a vector using the rapid DNA ligation kit (Roche). All other putative GGR genes were synthesized by GeneWiz (NJ, USA) and similarly cloned into the pET-24a vector at the same restriction sites. All gene constructs are available through the JBEI registry at http://public-registry.jbei.org (Table 1 and Additional file 1: Table S2).

**Table 2:** Specific activities of various enzymatic GGR reduction on geranylgeraniol and farnesol

	GGOH^a^	FOH^a^
Af GGR	22 (5)	9 (2)
MiGGR	10 (2)	8 (2)
PfGGR	9 (1)	7 (1)
TnGGR	20 (5)	5 (1)
SaGGR	50 (10)	8 (1)

^a^Units reported in nmol terpene units reduced mg^−1^ enzyme h^−1^

Ten nanogram of each plasmid was transformed by heat shock at 42 °C for 1 min into chemically competent *E. coli* BL21 cells harboring the pG-KJE8 plasmid encoding DnaK, DnaJ, GrpE, GroES, and GroEL protein chaperones (Takara Bio Inc., Shiga, Japan). Transformed cells were recovered in 1 mL of Lysogeny Broth (LB) medium (VWR) and incubated for 1 h at 37 °C with shaking at 200 rpm. Following recovery, cells were plated on LB Agar containing 50 mg/L of kanamycin (VWR) and 30 mg/L of chloramphenicol (VWR), and incubated overnight at 37 °C. Select colonies were grown overnight in LB medium containing 50 mg/L of kanamycin and 30 mg/L of chloramphenicol and stored in 20% glycerol (VWR) at − 80 °C for future use.

### Cell culture, protein expression, and protein purification

Overnight seed cultures of 1 mL each were inoculated into 400 mL of Terrific Broth (TB) medium supplemented with 50 mg/L kanamycin and 30 mg/L chloramphenicol and incubated at 37 °C and 200 rpm. At an OD_600_ of 0.2–0.3, chaperone overexpression was induced with 5 ng/mL tetracycline (VWR) and 2.5 mM arabinose (Sigma-Aldrich). After the OD_600_ reached ≥ 1.0, GGR expression was induced with 0.1 mM IPTG (VWR) and incubated at 18 °C overnight. Cells were pelleted at 6000×*g* for 10 min and immediately lysed using 20 mM phosphate buffer, pH 8.0 containing 1 mg/mL lysozyme, 20 mM imidazole, 200 mM NaCl, and 0.1 mM PMSF protease inhibitor (Sigma-Aldrich). After sonication for 10 min, the remaining cell debris was pelleted at 15,000×*g* for 45 min.

Protein expression was tested for each construct using SDS-PAGE and Western blot. For SDS-PAGE analysis, protein samples were normalized for concentration using absorbance at 280 nm. Lysates were diluted with 2× SDS loading dye buffer (Life Technologies, CA, USA) containing 10 mM DTT (Sigma-Aldrich) and incubated at 98 °C for 20 min. 10 µL of denatured lysate samples was loaded onto an 8–16% Tris–Glycine–SDS gradient gel (Bio-Rad), and separated using a voltage of 180 V in Tris–Glycine–SDS running buffer (Bio-Rad). Gels were either directly stained using GelCode Blue Safe Protein Stain (Thermo-Fisher) or transferred to a nitrocellulose membrane using the trans-Blot Turbo system (Life Technologies, CA, USA) for analysis by Western blot. Membranes were washed in TBS buffer (50 mM Tris, 150 mM NaCl, pH 7.4) and blocked overnight at 4 °C with 25 mL of 3% BSA in TBS-Tween20 (Sigma-Aldrich). The monoclonal mouse anti-His primary antibody (Sigma-Aldrich) was diluted 5000-fold, and an alkaline phosphatase-conjugated goat anti-mouse secondary antibody was diluted 10,000-fold in TBS-Tween20 containing 1% BSA. Membranes were incubated with antibodies for 1 h each at room temperature and washed three times in TBS-Tween20 after each antibody incubation. The membrane was then incubated in 10 mL of SigmaFast BCIP/NBT Alkaline Phosphatase detection solution (Sigma-Aldrich) for 10 min.

To further characterize those putative GGRs that showed significant protein expression, the cells harboring them were cultured in 400 mL of TB-Kan/Cm media and lysed as previously described. Their respective crude lysates were loaded directly onto a 1 mL HisTrap FastFlow column (GE Healthcare), washed with ten column volumes of 20 mM phosphate buffer containing 20 mM imidazole and 200 mM NaCl at pH 7.4, then eluted with the same buffer containing 240 mM imidazole. For enzyme kinetics, purified enzymes were buffer exchanged using 20 mM phosphate buffer at pH 7.4 and concentrated to 200–800 μM using 30 KDa molecular weight cutoff spin concentrators (EMD Millipore). Purified proteins were stored in 10% (v:v) glycerol and snap frozen in liquid nitrogen. Protein purity and sizes were verified by SDS-PAGE and protein concentrations were quantified by absorbance at 280 nm using each protein’s calculated extinction coefficient via the ExPASY ProtParam tool.

### In vitro enzyme kinetics assays

Validation of enzymatic substrate reduction was determined by incubating all assays in triplicate for each respective substrate and putative GGR for 1 h at 37 °C. All assays were performed at pH 7.4 in 100 mM sodium phosphate buffer containing 30–150 µM enzyme, 200 µM FAD (Sigma-Aldrich), and 65 mM sodium dithionite (Sigma-Aldrich). Standard assays for alcohol reduction were incubated with 100 µM enzyme and 500 µM (*E*,*E*)-farnesol (Alfa-Aesar) or (*E*,*E*,*E*)-geranylgeraniol (Sigma-Aldrich); pyrophosphate assays were performed at 100 µM FPP or GGPP (Sigma-Aldrich). Alcohol-based assays were quenched by liquid extraction using a 3:1 (v:v) LC-grade ethyl acetate solution containing 100 µM dodecanol as a GC internal standard (Sigma-Aldrich). The organic layer was extracted and stored at − 20 °C until analysis by GC–MS. Pyrophosphate assays were similarly quenched using LC-grade *n*-butanol (Sigma-Aldrich) 1:1 (v:v) and centrifuged at 15,000×*g* for 2 min. The *n*-butanol layer was dried for 45 min at ambient temperature using a Labconco speedvac, reconstituted in 25 µL of a 62:38 (v:v) acetonitrile/50 mM ammonium carbonate solution, and stored at − 20 °C until further analysis by LC–MS–TOF [33]. Characterization of enzymatic hydrolysis of isoprenoid pyrophosphate substrates by SaGGR and PfGGR was performed by quenching the enzyme reactions at 0, 2, 5, 10, 20, 40, and 60 min of incubation.

### Analysis of alcohol reduction by GC–MS

Product identification and quantification of farnesol and hydrofarnesol derivatives were modified from previous detection methods [47]. All GC–MS analyses were determined using an Agilent 6890 gas chromatography instrument coupled to an Agilent 5973 mass selective detector. 1 µL of extracted samples was injected in splitless mode onto an Agilent CycloSil-B column, with helium used as a carrier gas flowing at 1.0 mL/min. Following injection, the oven was held at 50 °C for 30 s, then increased to 175 °C at 35 °C/min. Farnesol and hydrofarnesols were resolved by increasing the temperature 4 °C/min up to 200 °C, then increased to 300 °C at a rate of 35 °C/min where it was held for 1.5 min. Geranylgeraniol and its hydrogenated derivatives were analyzed using the same injection method. After injection, the oven was held at 50 °C for 30 s, then increased to 235 °C at 35 °C/min. Hydrogeranylgeraniols were separated by increasing the oven temperature 4 °C/min to 250 °C, then ramped to 300 °C at a rate of 35 °C/min where it was held for 1.5 min.

The EI–MS detection was initiated after a solvent delay of 5.0 min. Detection and classification of hydrofarnesols were performed in scan mode at 9.8 scans/s ranging from 50 to 250 *m/z* in positive ion mode. For geranylgeraniol, the same scan parameters were implemented except for the mass range, which was expanded to 50–300 *m/z* in positive ion mode. The electron multiplier voltage was set to a gain factor of 1, with the MS ion source and quadrupole set to 230 °C and 150 °C, respectively.

Total ion chromatograms (TIC) were integrated using Agilent Technologies Masshunter software, version 6. Product formation was determined from the TIC area for C_15_ or C_20_ alcohol products eluting at each respective retention time. Absolute product concentrations were determined from standard curves (0–200 µM) of either farnesol or geranylgeraniol assuming the TIC area of each reduced product ionizes with an equivalent efficiency to that of the unreduced substrate (Additional file 1: Figure S1). Subsequently, enzyme turnover numbers for isoprenoid reduction were calculated as the total number of nanomoles of prenyl units reduced per milligram of enzyme in 1 h.

### Analysis of pyrophosphate reduction by LC–MS–TOF

The separation of FPP, GGPP, and their reduced forms was conducted on a ZIC-pHILIC column (150 mm length, 2.1 mm internal diameter, and 5 µm particle size, Merck) using an Agilent Technologies 1200 Series Rapid Resolution high-performance liquid chromatography (HPLC) system. Solvents for HPLC were purchased from HoneyWell and were of HPLC grade or higher. The mobile phases used for this analysis were (A) 50 mM ammonium carbonate (Fluka, 30–33% NH_3_ basis) in water and (B) acetonitrile. Analytes were eluted isocratically with a mobile phase composition of 62% B at a flow rate of 0.2 mL/min. The total run time of the method was 6.5 min. The temperature of the sample tray was maintained at 6 °C using an Agilent FC/ALS Thermostat. The column compartment was set to 40 °C. A sample injection volume of 2 µL was used throughout [33].

The HPLC system was coupled to an Agilent Technologies 6210 time-of-flight mass spectrometer (LC–TOF–MS) by a 1/3 post-column split. Contact between both instrument set-ups was established using a LAN card to trigger the MS into operation upon the initiation of a run cycle from the MassHunter workstation (Agilent Technologies). Electrospray ionization (ESI) was conducted in the negative ion mode and a capillary voltage of − 3500 V was utilized. MS experiments were carried out in full scan mode, at 0.86 spectra/second for the detection of [M−H]^−^ ions. The instrument was tuned for a range of 50–1700 *m/z*. Prior to LC–TOF–MS analysis, the TOF–MS was calibrated via an ESI-L low concentration tuning mix (Agilent Technologies).

Data acquisition and processing were performed by the Agilent Technologies MassHunter software package. Product formation was determined using extracted ion chromatogram abundances (± 0.02 Da) for each molecule’s [M−H]^−^ mass (Additional file 1: Table S1). Substrate and product hydrolysis of SaGGR and PfGGR was characterized as a function of time by measuring the relative ratios of prenyl pyrophosphates (FPP/GGPP and reduced products) and monophosphates (FP/GGP and reduced products) at quenched fractions collected at 0, 2, 5, 10, 20, 40, and 60 min. Relative reductase reactivity among GGRs was determined by measuring the fractional abundance of singly, doubly, or triply reduced products to the total ion abundance present for intact and hydrolyzed moieties [33]. Integrated areas for hydrolyzed monophosphate products were assumed to have the same ionization intensities as their pyrophosphate counterparts, as determined by their standard curves measured from 0 to 120 µM (Additional file 1: Figure S6).

## Additional file

**Additional file 1: Figure S1.** TIC for neat GGOH (RT = 8.4 min, top) and FOH (RT = 8.0 min, middle) substrates. The standard curve for quantifying farnesol (circles) and geranylgeraniol (squares) by GC–MS (bottom) exhibited a linear response for both substrates between 0 and 200 µM. **Figure S2.** Verification of accelerated substrate reduction as a function of enzyme concentration for GGOH (left) and FOH (right) for the Af (circles), Mi (squares), Tn (filled triangles), Sa (filled upside down triangles), and Pf (unfilled triangles) GGR enzymes. Specific activities are quoted in Table 1. **Figure S3.** Comparison of mass spectra between a side product containing one internal prenyl group reduced within H_4_-GGOH with an 8.0 min retention time (Top, black) and the assigned product with the terminal prenyl group reduced in H_4_-GGOH eluting at 7.7 min (Bottom, green). **Figure S4.** (Top) normalized TIC of farnesol activity assay incubated for 2 h with SaGGR at 50 °C, pH 5.5 showing a modest abundance of fully reduced farnesol (RT = 7.0 ± 0.1 min). For reference, FOH and H_4_-FOH elute at retention times of 7.9 and 7.3 min, respectively. H_2_-FOH (RT = 7.5 min) was not observed in any quantifiable abundance. All substrate and cofactor concentrations were held constant. **Figure S5.** Comparison of mass spectra between the middle prenyl group reduced within the putative H_2_-FOH side product eluting at 7.8 min retention time (Top, black) and the assigned product with the terminal prenyl group reduced in H_2_-FOH eluting at 7.6 min (Bottom, green). **Figure S6.** Standard curve for quantifying FPP (circles) and GGPP (squares) by LC–MS–TOF. **Figure S7.** MS-TOF Spectrum of 100 µM FPP and GGPP standards (Top). (Bottom) relative abundances of GGPP and GGP (left) or FPP and FP (middle) after incubation under standard assay conditions; negative controls containing all assay components without enzyme (right) rule out the possibility of spontaneous hydrolysis of substrate, as the ratio of pyrophosphate (dark gray) to monophosphate (light gray) products remain constant as a function of time. Reduced products within each mass grouping are included in the total abundance. **Figure S8.** Demonstration that first-order substrate hydrolysis catalyzed by either SaGGR (squares) or PfGGR (triangles) in either FPP (left) or GGPP (right). The no enzyme control (circles) contained all assay components except enzyme. **Figure S9.** Timecourse comparison of the standard assay for SaGGR (top row) and PfGGR (bottom row) on either GGPP or FPP substrates. Pyrophosphate abundances are shown in the left column and monophosphate abundances are shown in the right column under each substrate. Products are expressed as having zero reductions (blue), one reduction (green), two reductions (red), or three reductions (orange). **Table S1.** Masses used to analyze various products formed from GGR standard assays incubated with prenyl pyrophosphates. ^a^Masses reported are for the deprotonated [M−H]^−^ parent ion in negative mode detection. **Table S2.** Table of plasmids used in the present study. The strains harboring individual plasmid are available at the public registry of the Joint BioEnergy Institute (https://public-registry.jbei.org/) under the ID’s listed in the righthand column.

## Abbreviations

GPP: geranyl pyrophosphate
FPP: farnesyl pyrophosphate
GGPP: geranylgeranyl pyrophosphate
FOH: farnesol
GGOH: geranylgeraniol
GGR: geranylgeranyl reductase
LC–MS: liquid chromatography–mass spectrometry
TOF: time of flight
GC–MS: gas chromatography–mass spectrometry
TIC: total ion chromatogram
