# Supplementary material for: Discovery of novel geranylgeranyl reductases and characterization of their substrate promiscuity
Source: Biotechnol Biofuels. 2018 Dec 28;11:340. doi: 10.1186/s13068-018-1342-2 (PMC6309074; doi:10.1186/s13068-018-1342-2)
Supplement: Supplementary file 1 — Additional file 1: Figure S1. TIC for neat GGOH (RT = 8.4 min, top) and FOH (RT = 8.0 min, middle) substrates. The standard curve for quantifying farnesol (circles) and geranylgeraniol (squares) by GC–MS (bottom) exhibited a linear response for both substrates between 0 and 200 µM. Figure S2. Verification of accelerated substrate reduction as a function of enzyme concentration for GGOH (left) and FOH (right) for the Af (circles), Mi (squares), Tn (filled triangles), Sa (filled upside down triangles), and Pf (unfilled triangles) GGR enzymes. Specific activities are quoted in Table 1. Figure S3. Comparison of mass spectra between a side product containing one internal prenyl group reduced within H4-GGOH with an 8.0 min retention time (Top, black) and the assigned product with the terminal prenyl group reduced in H4-GGOH eluting at 7.7 min (Bottom, green). Figure S4. (Top) normalized TIC of farnesol activity assay incubated for 2 h with SaGGR at 50 °C, pH 5.5 showing a modest abundance of fully reduced farnesol (RT = 7.0 ± 0.1 min). For reference, FOH and H4-FOH elute at retention times of 7.9 and 7.3 min, respectively. H2-FOH (RT = 7.5 min) was not observed in any quantifiable abundance. All substrate and cofactor concentrations were held constant. Figure S5. Comparison of mass spectra between the middle prenyl group reduced within the putative H2-FOH side product eluting at 7.8 min retention time (Top, black) and the assigned product with the terminal prenyl group reduced in H2-FOH eluting at 7.6 min (Bottom, green). Figure S6. Standard curve for quantifying FPP (circles) and GGPP (squares) by LC–MS–TOF. Figure S7. MS-TOF Spectrum of 100 µM FPP and GGPP standards (Top). (Bottom) relative abundances of GGPP and GGP (left) or FPP and FP (middle) after incubation under standard assay conditions; negative controls containing all assay components without enzyme (right) rule out the possibility of spontaneous hydrolysis of substrate, as the ratio of pyrophosphate (dark gra [file 13068_2018_1342_MOESM1_ESM.docx]

**Additional file 1**

**Discovery of novel geranylgeranyl reductases and characterization of their substrate promiscuity**

Corey W. Meadows^1,2,†^, Florence Mingardon^3,†^, Brett M. Garabedian^1,2^, Edward E. K. Baidoo^1,2^, Veronica T. Benites^1,2^, Andria V. Rodrigues^1,2^, Raya Abourjeily^3^, Angelique Chanal^3^, Taek Soon Lee^1,2,*^

^1^Joint BioEnergy Institute, 5885 Hollis Street, Emeryville, CA 94608, USA.

^2^Biological Systems & Engineering Division, Lawrence Berkeley National Laboratory, Berkeley, CA 94720, USA.

^3^Total Raffinage Chimie, 2 Pl. Jean Millier, 92400 Courbevoie, France

^†^These authors contributed equally to this work.

^*^Corresponding author: Dr. Taek Soon Lee, Joint BioEnergy Institute, 5885 Hollis St. 4^th^ floor, Emeryville, CA 94608, USA; Phone: +1-510-495-2470, Fax: +1-510-495-2629, E-mail: tslee@lbl.gov

**Figure S1.** TIC for neat GGOH (RT = 8.4 minutes, top) and FOH (RT = 8.0 minutes, middle) substrates. The standard curve for quantifying farnesol (circles) and geranylgeraniol (squares) by GC-MS (bottom) exhibited a linear response for both substrates between 0-200 µM.

**
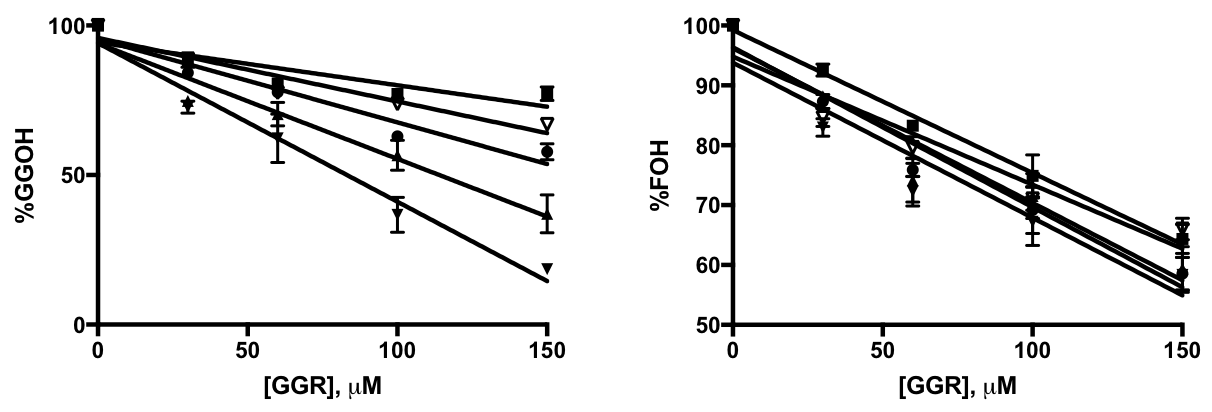
**

**Figure S2.** Verification of accelerated substrate reduction as a function of enzyme concentration for GGOH (left) and FOH (right) for the Af (circles), Mi (squares), Tn (filled triangles), Sa (filled upside down triangles), and Pf (unfilled triangles) GGR enzymes. Specific activities are quoted in Table 2.

**
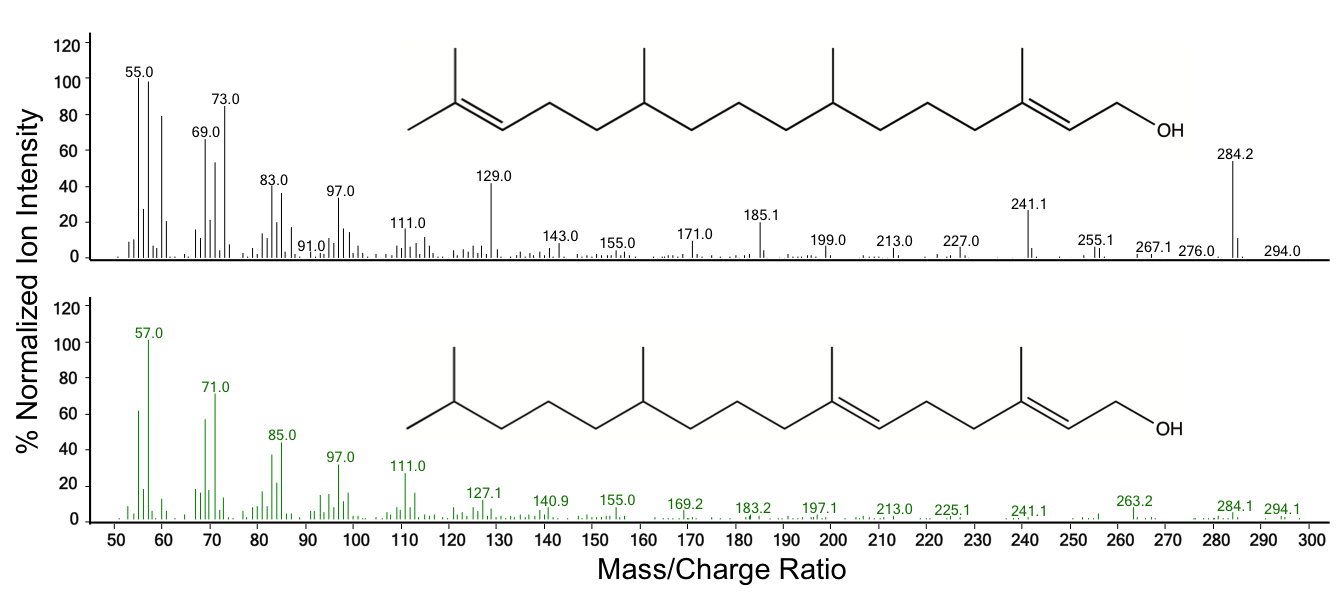
**

**Figure S3.** Comparison of mass spectra between a side product found after incubation with PfGGR, where the terminal prenyl group remains oxidized within H_4_-GGOH with an 8.0 minutes retention time (Top, black) and the assigned product with the terminal prenyl group reduced in H_4_-GGOH eluting at 7.7 minutes (Bottom, green). The structures are suggested from NIST database.

**
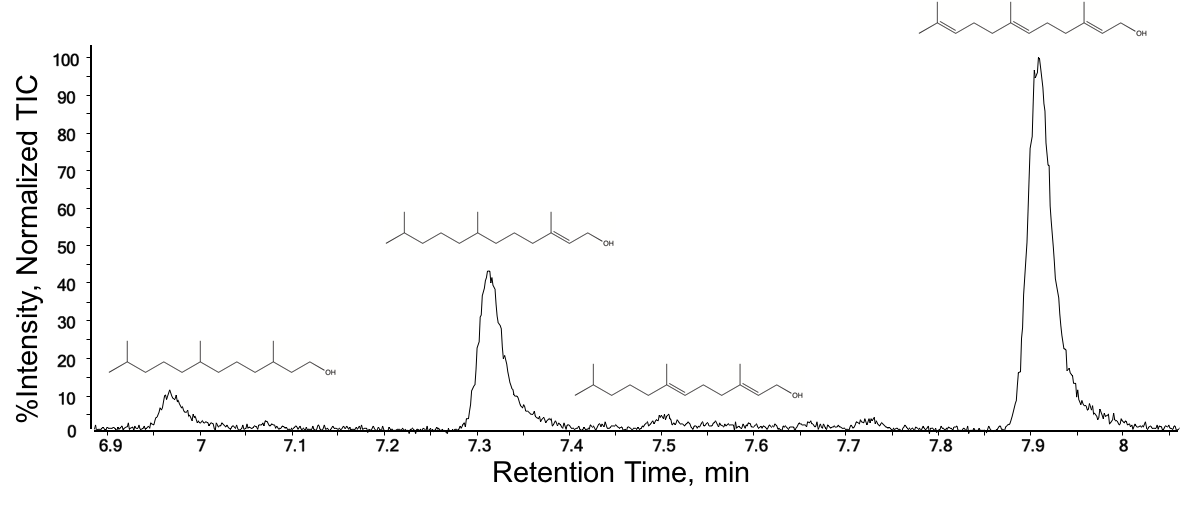
**

**Figure S4.** Normalized TIC of farnesol incubated for 2 hours with SaGGR at 50^°^C, pH 5.5 showing a modest abundance of fully reduced farnesol (RT = 7.0 ± 0.1 minutes). For reference, FOH and H_4_-FOH elute at retention times of 7.9 and 7.3 minutes, respectively. H_2_-FOH (RT = 7.5 minutes) was not observed in any quantifiable abundance. All substrate and cofactor concentrations were held constant.

**
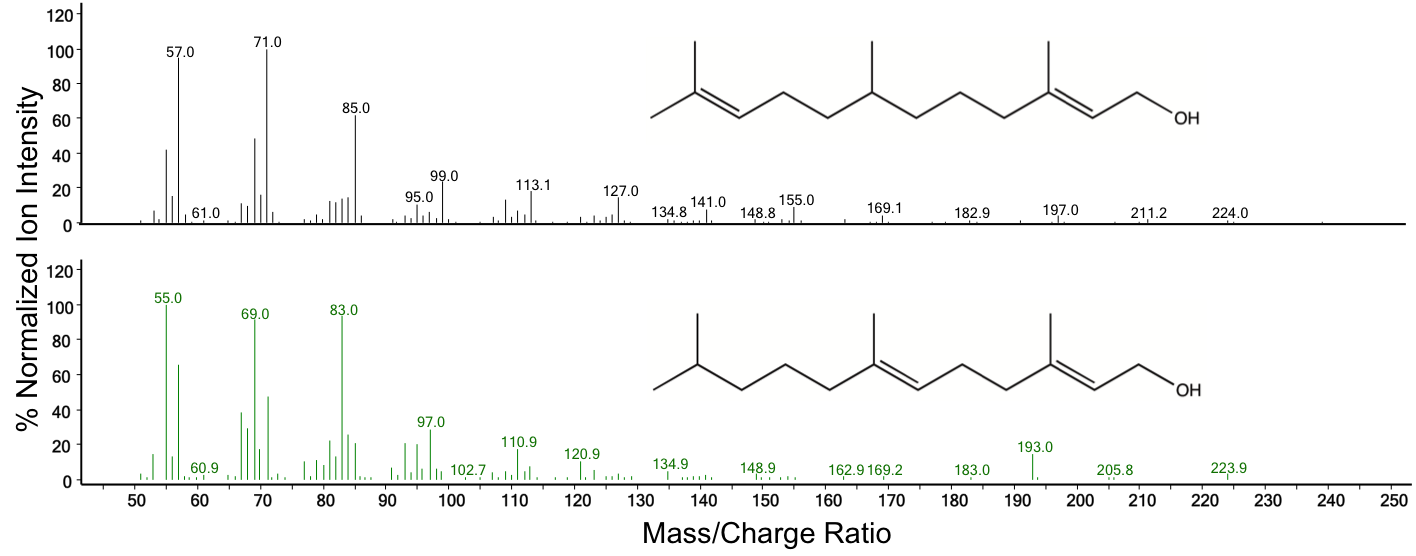
**

**Figure S5.** Comparison of PfGGR product profiles. Mass spectra between the middle prenyl group reduced within the putative H_2_-FOH side product eluting at 7.8 minutes retention time (Top, black) and the assigned product with the terminal prenyl group reduced in H_2_-FOH eluting at 7.6 minutes (Bottom, green).

**Figure S6.** Standard curve for quantifying FPP (circles) and GGPP (squares) by LC-MS-TOF. Error bars are contained within each data point.

**
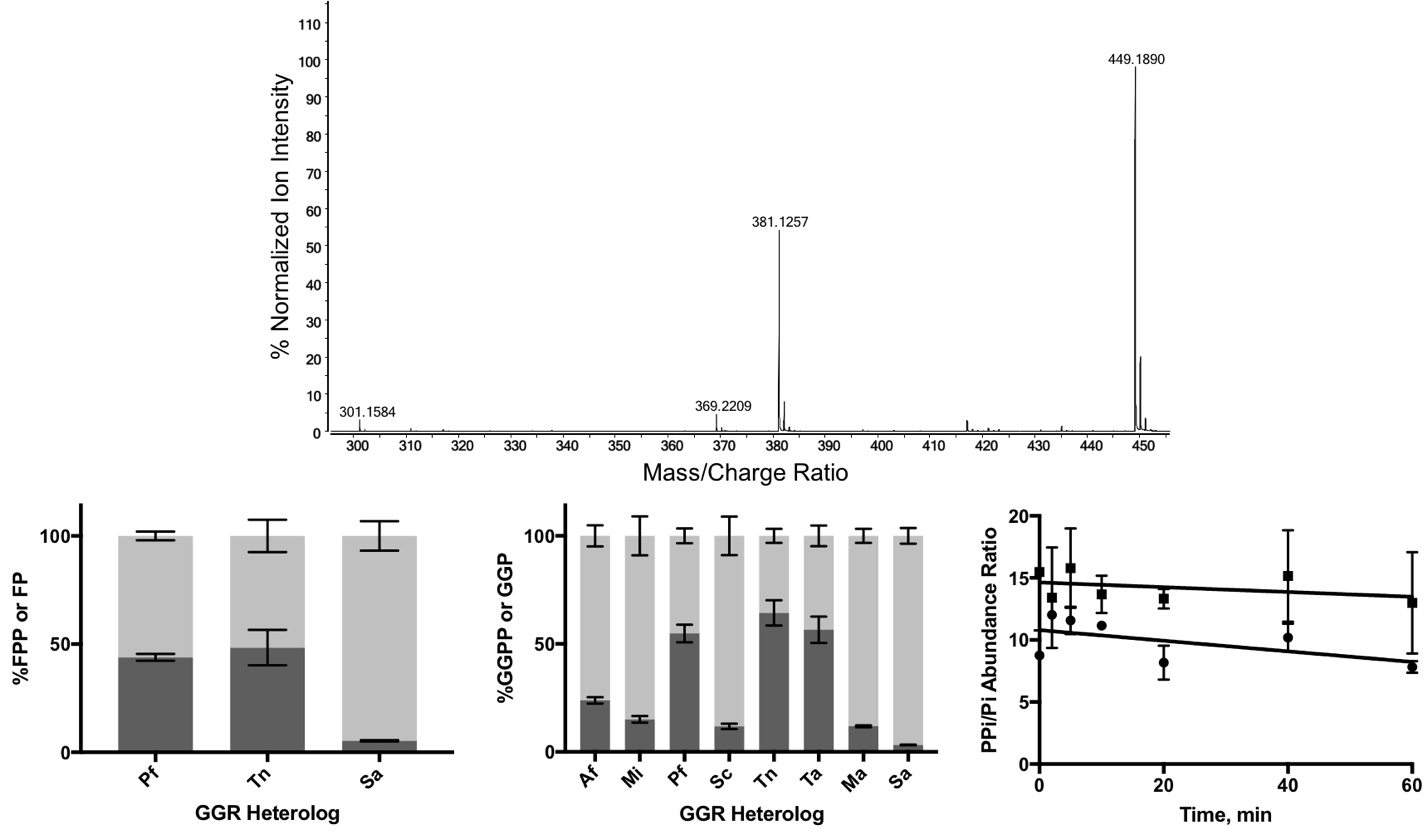
**

**Figure S7.** MS-TOF Spectrum of 100 µM FPP and GGPP standards (Top). (Bottom) Relative abundances of FPP and FP (left) or GGPP and GGP (middle) after incubation under standard assay conditions; negative controls containing all assay components without enzyme (right) rule out the possibility of spontaneous hydrolysis of substrate, as the ratio of pyrophosphate (dark gray) to monophosphate (light gray) products remain constant as a function of time. Reduced products within each mass grouping are included in the total abundance.

**
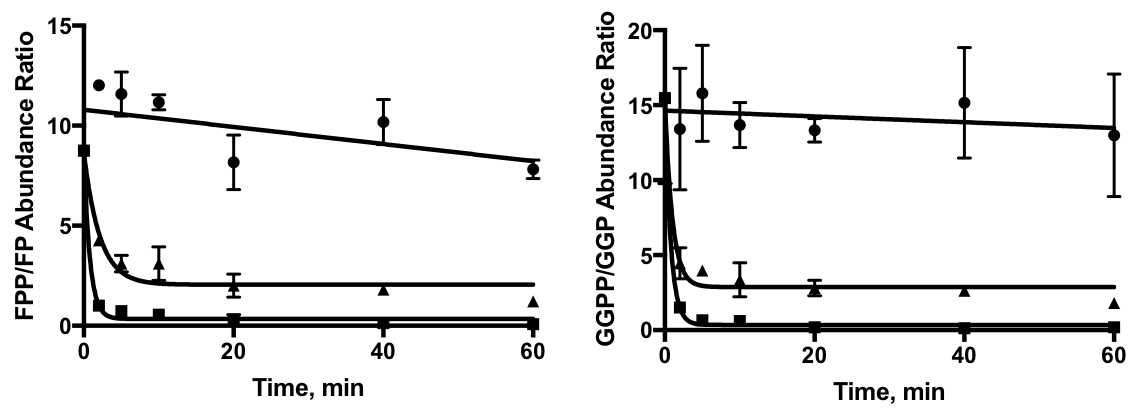
**

**Figure S8.** Demonstration that first-order substrate hydrolysis catalyzed by either SaGGR (squares) or PfGGR(triangles) in either FPP (left) or GGPP (right). The no enzyme control (circles) contained all assay components except enzyme.


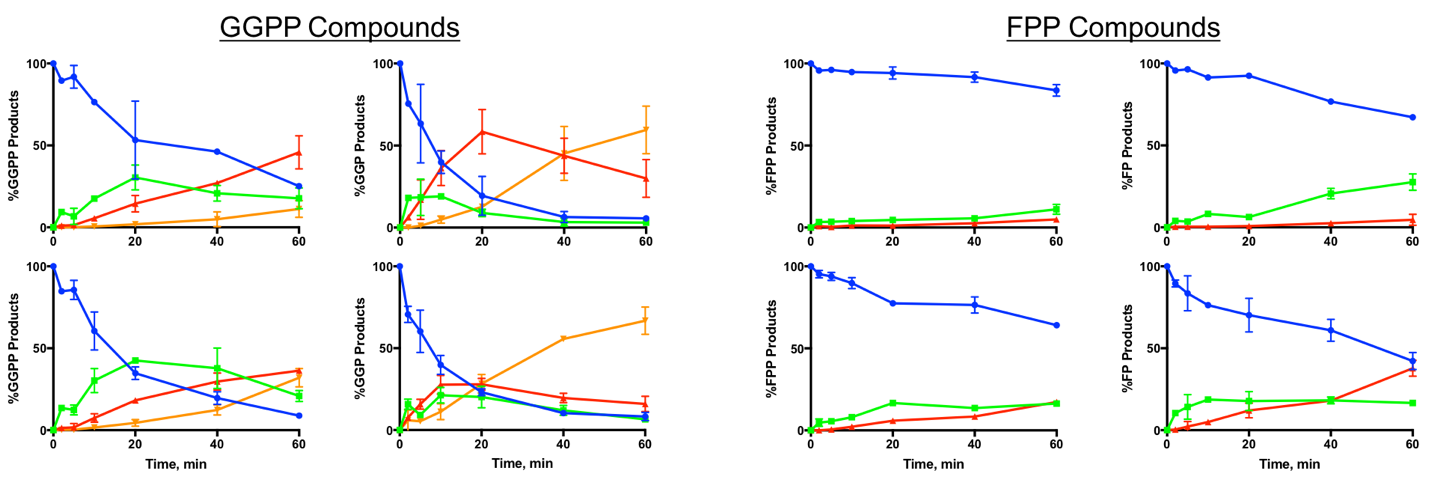


**Figure S9.** Time course comparison of the standard assay for SaGGR (top row) and PfGGR (bottom row) on either GGPP or FPP substrates. Pyrophosphate abundances are shown in the left column and monophosphate abundances are shown in the right column under each substrate. Products are expressed as having zero reductions (blue), one reduction (green), two reductions (red), or three reductions (orange).

**Table S1.** Masses used to analyze various products formed from GGR standard assays incubated with prenyl pyrophosphates. *^a^*Masses reported are for the deprotonated [M-H]^-^ parent ion in negative mode detection.

| **C_20_ Pyrophosphates** | | | **C_20_ Monophosphates** | | |
| --- | --- | --- | --- | --- | --- |
| Compound | Formula | Molecular Mass, Da*^a^* | Compound | Formula | Molecular Mass, Da*^a^* |
| GGPP | C_20_H_36_O_7_P_2_ | 449.18635 | GGP | C_20_H_35_O_4_P | 369.22002 |
| H_2_-GGPP | C_20_H_38_O_7_P_2_ | 451.202 | H_2_-GGP | C_20_H_37_O_4_P | 371.23567 |
| H_4_-GGPP | C_20_H_40_O_7_P_2_ | 453.21765 | H_4_-GGP | C_20_H_39_O_4_P | 373.25132 |
| H_6_-GGPP | C_20_H_42_O_7_P_2_ | 455.2333 | H_6_-GGP | C_20_H_41_O_4_P | 375.26697 |
| H_8_-GGPP | C_20_H_44_O_7_P_2_ | 457.24895 | H_8_-GGP | C_20_H_43_O_4_P | 377.28262 |
| **C_15_ Pyrophosphates** | | | **C_15_ Monophosphates** | | |
| Compound | Formula | Molecular Mass, Da*^a^* | Compound | Formula | Molecular Mass, Da*^a^* |
| FPP | C_15_H_28_O_7_P_2_ | 381.1237 | FP | C_15_H_27_O_4_P | 301.1574 |
| H_2_-FPP | C_15_H_30_O_7_P_2_ | 383.1394 | H_2_-FP | C_15_H_29_O_4_P | 303.17307 |
| H_4_-FPP | C_15_H_32_O_7_P_2_ | 385.155 | H_4_-FP | C_15_H_31_O_4_P | 305.18872 |
| H_6_-FPP | C_15_H_34_O_7_P_2_ | 387.1707 | H_6_-FP | C_15_H_33_O_4_P | 307.20437 |

**Table S2.** Table of plasmids used in the present study. The strains harboring individual plasmid are available at the public registry of the Joint BioEnergy Institute (https://public-registry.jbei.org/) under the ID’s listed in the righthand column.

| Plasmid Name | Organism | Ref. in JBEI registry |
| --- | --- | --- |
| pET-AfGGR | *Archaeoglobus fulgidus #1* | JBx_095992 |
| pET-Af2GGR | *Archaeoglobus fulgidus#2* | JBx_095994 |
| pET-AtGGR | *Arabidopsis thaliana* | JBx_095980 |
| pET-CnGGR | *Candidatus Nitrosopumilus* | JBx_095996 |
| pET-CtGGR | *Corynebacterium terpenotabidum* | JBx_095998 |
| pET-GpGGR | *Gordonia polyisoprenivorans* | JBx_096000 |
| pET-HcGGR | *Halorubrum californiensis* | JBx_096002 |
| pET-HlGGR | *Halostagnicola larsenii X* | JBx_096004 |
| pET-HsGGR | *Haloterrigena salina* | JBx_096006 |
| pET-Hv1GGR | *Haloferax volcanii #1* | JBx_096008 |
| pET-Hv2GGR | *Haloferax volcanii #2* | JBx_096010 |
| pET-Ma1GGR | *Methanosarcina acetivorans #1* | JBx_096050 |
| pET-Ma2GGR | *Methanosarcina acetivorans #2* | JBx_096012 |
| pET-Ma3GGR | *Methanosarcina acetivorans #3* | JBx_096014 |
| pET-MbGGR | *Methanococcoides burtonii* | JBx_096020 |
| pET-McGGR | *Metallosphaera cuprina* | JBx_096022 |
| pET-MiGGR | *Methanocaldococcus infernus* | JBx_096024 |
| pET-MmGGR | *Methanococcus maripaludis* | JBx_096026 |
| pET-Mr1GGR | *Methanobrevibacter ruminantium #1* | JBx_096028 |
| pET-Mr2GGR | *Methanobrevibacter ruminantium #2* | JBx_096030 |
| pET-NgGGR | *Nitrososphaera gargensis* | JBx_096032 |
| pET-PcfGGR | *Pyrococcus furiosus* | JBx_096034 |
| pET-PfGGR | *Pyrolobus fumarii* | JBx_096036 |
| pET-SaGGR | *Sulfolobus acidocaldarius* | JBx_095970 |
| pET-ScGGR | *Streptomyces coelicolor #1* | JBx_096038 |
| pET-SeGGR | *Synechococcus elongatus #1* | JBx_096040 |
| pET-SynGGR | *Synechocystis species* | JBx_096042 |
| pET-TaGGR | *Thermoplasma acidophilum #1* | JBx_096052 |
| pET-TnGGR | *Thermococcus nautili* | JBx_096044 |
| pET-Tr1GGR | *Thermocrinis ruber #1* | JBx_096046 |
| pET-Tr2GGR | *Thermocrinis ruber #2* | JBx_096048 |
